# Neurons in the primary visual cortex of freely moving rats encode both sensory and non-sensory task variables

**DOI:** 10.1371/journal.pbio.3002384

**Published:** 2023-12-04

**Authors:** Anqi Zhang, Anthony M. Zador

**Affiliations:** 1 Cold Spring Harbor Laboratory, Cold Spring Harbor, New York, United States of America; 2 Cold Spring Harbor Laboratory School of Biological Sciences, Cold Spring Harbor, New York, United States of America; Center for Brain Research, Medical University of Vienna, AUSTRIA

## Abstract

Neurons in primary visual cortex (area V1) are strongly driven by both sensory stimuli and non-sensory events. However, although the representation of sensory stimuli has been well characterized, much less is known about the representation of non-sensory events. Here, we characterize the specificity and organization of non-sensory representations in rat V1 during a freely moving visual decision task. We find that single neurons encode diverse combinations of task features simultaneously and across task epochs. Despite heterogeneity at the level of single neuron response patterns, both visual and nonvisual task variables could be reliably decoded from small neural populations (5 to 40 units) throughout a trial. Interestingly, in animals trained to make an auditory decision following passive observation of a visual stimulus, some but not all task features could also be decoded from V1 activity. Our results support the view that even in V1—the earliest stage of the cortical hierarchy—bottom-up sensory information may be combined with top-down non-sensory information in a task-dependent manner.

## Abstract 2

Neurons in primary visual cortex (area V1) are strongly driven by both sensory stimuli and nonsensory events. This study shows that during visual and auditory decisions, rat V1 represents multiple task features in an overlapping and distributed fashion, with some modulation by task demands.

## Introduction

The brain processes and transforms sensory inputs to generate appropriate motor outputs. How brain areas contribute to this goal is related to the features they can represent. In primary visual cortex, neural activity has historically been characterized in terms of stimulus parameters such as orientation, spatial frequency, temporal frequency, and direction of visual motion [1–3]. By contrast, complex combinations of task-relevant and abstract features are often found in downstream areas in parietal and frontal cortices [4–8]. Although it has long been recognized that sensory cortices are not driven solely by bottom-up sensory inputs—the first single unit recordings reported attentional modulation of auditory responses in the cat [9]—there has recently been growing recognition of the importance of non-sensory responses in primary visual cortex (V1), such as those related to locomotion, arousal, and body movements [10–12].

The role of non-sensory responses in primary sensory cortices remains an open question. Although sensory representations in primary sensory cortices are important for perceptual decisions, the magnitude of stimulus-evoked activity in sensory cortices is frequently overshadowed by the magnitude of activity due to task-condition, movement, or outcome [10,11,13–16]. Non-sensory signals both modulate and appear independently of sensory-related activity in primary visual and auditory cortices [10,11,15–20]. In V1, non-sensory representations may support some visual computations, such as computing visual expectations during virtual reality locomotion or navigation, and in these cases are coherent with relevant sensory representations [19,21–23]. However, non-sensory–driven activity has also been observed when such computations are not necessary and can both correlate with and occur independently of task variables [10]. We set out to understand how task-related non-sensory activity is organized and how it relates to sensory encoding and task demands.

Here, we used extracellular methods to record responses from single neurons in area V1 of freely moving rats performing a visual decision task. We find that neurons in this area encode both sensory and non-sensory task variables. In control animals trained to perform a similar but nonvisual task, the encoding of sensory stimuli was similar, but the fidelity with which some non-sensory task variables were encoded was markedly diminished. Our results demonstrate that even in V1—the earliest stage of the cortical hierarchy—bottom-up sensory information is combined with top-down information in a task-dependent manner.

## Results

In what follows, we first describe a visual spatial decision-making paradigm for freely moving rats, along with software methods to constrain the animal’s viewing position and angle. Then, we characterize visual and nonvisual representations in V1 single neuron activity recorded using tetrode microdrives. We analyze this activity for representations of task parameters such as stimulus, choice, movement parameters, and outcome. We then investigate whether neurons are specialized for encoding single task features or are influenced by combinations of task features within and across task epochs. Conversely, we ask to what extent these task features can be read out from neural populations at various points in the task. Finally, we compare V1 response profiles during visual spatial decisions to those during an analogous but visually independent task.

### “Cloud of dots” visual decision-making task

**Fig 1:**
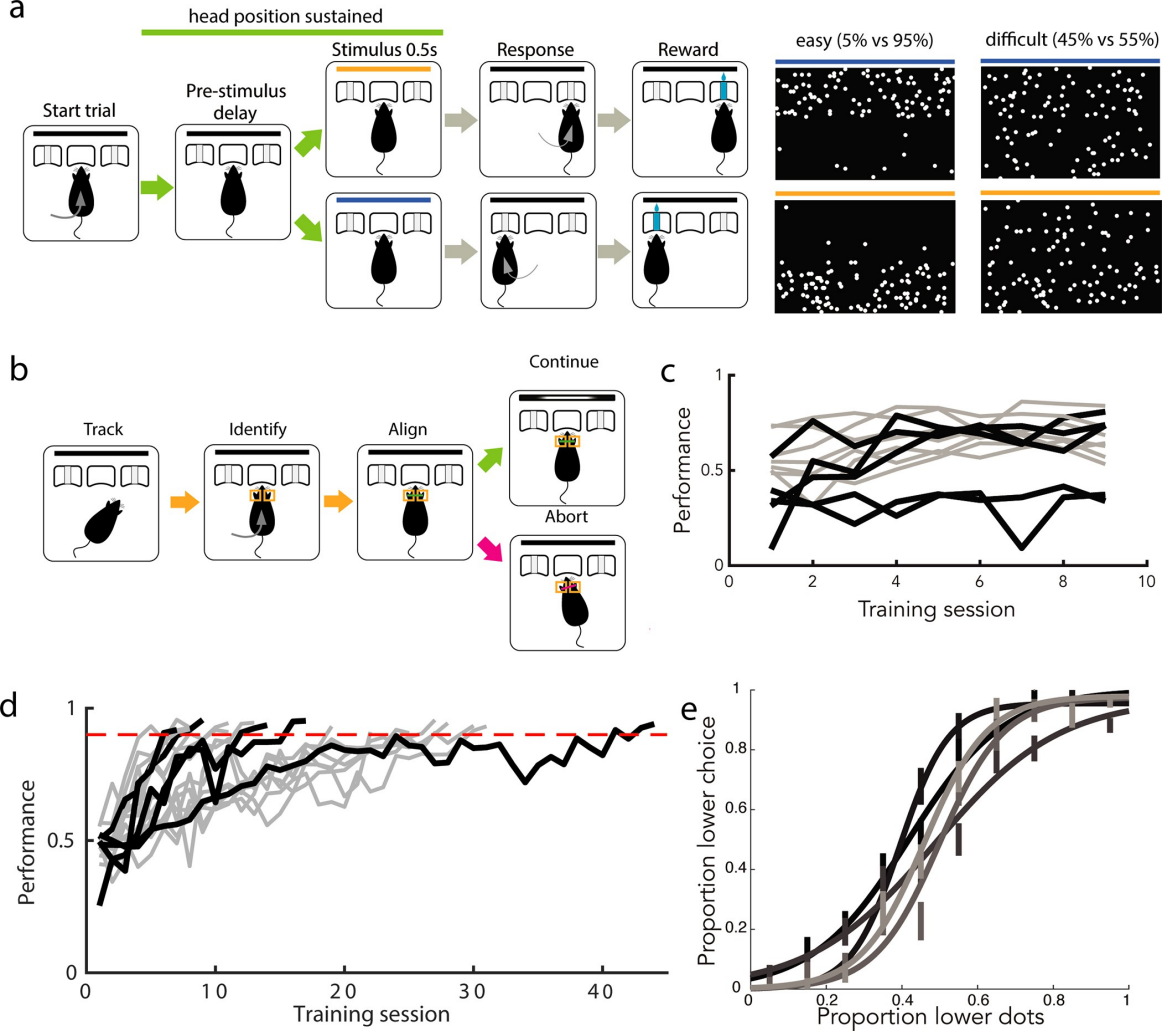
Rats reliably learn a “cloud of dots” visual decision-making task. (a) Task design, with example stimulus frames for upper hemifield (top) and lower hemifield (bottom) trials (left: easy, right: difficult). Stimulus duration is 0.5 s; all other task epochs have variable duration. (b) Virtual head fixation algorithm, condition is active for portion of trial marked by green line in (a). (c) Proportion of trials completed increased as animals were trained on head fixation. Across animals trained on head fixation after learning the visual rule, the mean proportion of completed trials on day 1 of training was 0.483; this increased to 0.635 by day 9. Black trajectories denote animals whose neural recordings were included in this dataset, gray trajectories denote animals who were trained but no recordings were performed. (d) Animals typically reached stable performance above 90% accuracy on completed easy trials in fewer than 30 sessions (median = 16 sessions, +/−10 std). Color scheme as in (c). (e) Psychometric performance on single sessions after reaching performance criterion on easy stimuli, prior to neural recordings, for each animal included in neural dataset. Error bars indicate standard error of the mean. The underlying data for this figure are available for download from 10.17632/5ms7gcb67j.1.

To probe the patterns of representations in primary visual cortex during a freely moving visually guided behavior, we first designed a fixed-time visual decision-making task for freely moving rats. Rats were placed into a behavior chamber containing 3 nosepokes [14,24]. Rats self-initiated trials by poking into the center stimulus viewing port and were presented with a 500 ms-long visual stimulus of distributed flickering dots (Fig 1A). They were asked to judge the region of higher dot density (top versus bottom) presented in the stimulus and reported their decision by poking into one of the side nosepokes after delivery of a decision tone signaling the beginning of the decision period. Correct choices earned a small water reward, while incorrect choices earned a punishment tone and time-out.

The spatially distributed stimuli were designed to exploit the retinotopic organization in V1, but neural responses would only be interpretable if the stimulus could be oriented in a reproducible manner with respect to the animal’s visual field over trials. We therefore additionally required animals to fulfill a head position criterion prior to and throughout the duration of stimulus delivery. We did not control for eye position because we reasoned that the small amplitude eye movements made by rats, which are reduced further when the head is stationary [25], would not impact the low spatial resolution (upper versus lower) at which animals were required to discriminate. Instead of a physical head fixation protocol [26], we developed a noninvasive software-based method to virtually constrain the viable head positions at the stimulus viewing port (Fig 1B). We used Bonsai open source software to continuously acquire and segment online video of the behavior chamber [27]. Upon trial initiation by the animal, we measured the size and relative position of the animal’s ears in predefined regions of interest (adjusted on a per-animal basis). As long as both size and distance criteria (in both x and y dimensions) were met, the trial was allowed to continue. If any criterion was violated prior to or during stimulus presentation, the trial was aborted and a short time out was delivered. We trained animals to fulfill this postural criterion immediately following acquisition of the decision rule. Rats learned to adjust their head position over the first few sessions of head position training, improving their proportion of successfully completed trials (Fig 1C).

We trained 17 rats to perform this task, reaching a level of 90%+ accuracy on easiest trials over the course of 16 (median, IQR = 16.75) sessions. Of these, 12 animals were trained to maintain head position, and recordings in V1 were made from 5 of these animals. Choice accuracy varied with stimulus difficulty, producing psychometric behavior within and across sessions (Fig 1E).

### Diversity of responses in primary visual cortex during visually guided decisions

**Fig 2:**
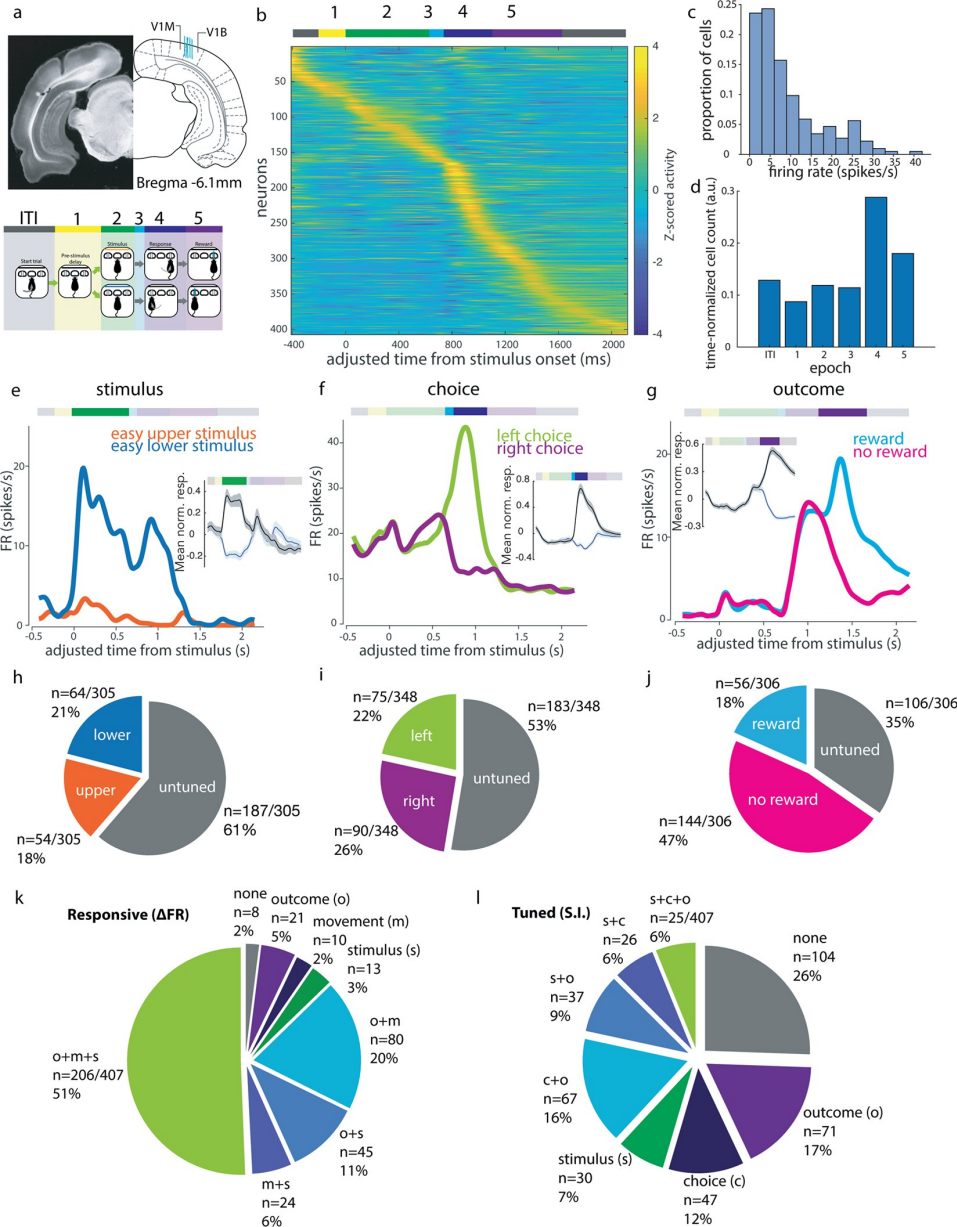
Tuned representations of several task features during visual decisions by V1 single neurons. (a) Recording sites (blue lines each represent tetrode bundle center in one animal) and definitions of task epochs used in analysis. Schematic adapted from Paxinos and Watson [28]. V1M: primary visual cortex (monocular); V1B: primary visual cortex (binocular); V2L: secondary visual cortex, lateral area; V2MM: secondary visual cortex, mediomedial area; V2ML: secondary visual cortex, mediolateral area; ITI: intertrial interval. (b) Mean trial-aligned z-scored activity for all single units in the cloud of dots task (*N* = 5, *n* = 407) spans the duration of the trial. Adjusted time aligns all trials to the same time axis to allow pooling of variable length epochs (see Methods). Task epochs as denoted by colored bar above. (c) Distribution of mean firing rates of putative single units over the session. (d) Proportion of single units displaying peak activity in each epoch, normalized to the mean duration of each epoch. (e–g) Example neurons showing (e) stimulus, (f) choice, and (g) outcome tuning within the respective epochs. Insets show mean normalized response amplitudes (z-scored activity) over all such selective neurons (black indicates preferred, blue indicates non-preferred, shaded area indicates SEM). (h) Proportion of visual location tuned cells in recording dataset. (i) Proportion of choice direction tuned cells. (j) Proportion of reward tuned cells. (k) Proportions of cells with significant modulation of activity (paired *t* test of epoch rates within trials, *p* < 0.05) during stimulus (s, green), movement (m, blue), or outcome (o, purple) epochs compared to pre-stimulus baseline (epoch 1 from panel b). (l) Proportion of all single units (*n* = 407) tuned to some combination of stimulus (s), choice (c), and outcome (o) across epochs. The underlying data for this figure are available for download from 10.17632/5ms7gcb67j.1.

We used 32-channel tetrode drives to record putative single unit activity in V1 during this visually guided (Fig 2A) decision task in order to understand the extent and specificity of task-related information available to this early stage in the visual pathway. We recorded neuronal responses in V1 from 516 units in 5 rats. In what follows, we analyze responses from well-isolated single units (*n* = 407), defined as those with consistent, large-amplitude waveforms and fewer than 1% ISI violations. The peak mean activity of an individual unit could occur at any point during the trial, with an enrichment of units showing maximum activity during the movement epoch (Fig 2B and 2D). The activity patterns were similar in multiunit activity (*n* = 109, S1 Fig).

We first quantified the tuning properties of single units to sensory and non-sensory task features during different task epochs. For each epoch of interest, we limited this analysis to single units firing more than 1 spike/s on average during that behavioral epoch. As a result, the set of single units included for each epoch differed slightly (for example, a neuron that fired during stimulus presentation but was silent during movement would be included in stimulus epoch tuning analyses but not movement epoch tuning analyses; see Methods for details). For each feature of interest (stimulus identity, choice side, outcome), we defined a selectivity index (si) to compare the activity evoked by different conditions within a given task epoch:

$$si=\frac{{FR}_{condition\_A}-{FR}_{condition\_B}}{{FR}_{condition\_A}+{FR}_{condition\_B}}$$ (1)

where conditions A and B refer to the 2 conditions being compared. In the case of stimulus selectivity, for example, FR_easy lower stimulus_ refers to the firing rate for the 0.5 s following stimulus onset when an easy lower stimulus was presented. Comparing the observed selectivity indices to the distribution of indices calculated from the shuffled label control, we identified 39% (118/305) of the single units that were active during the stimulus epoch as significantly stimulus selective (Fig 2H).

We also observed many neurons with above-baseline activity during task epochs other than the stimulus epoch (Fig 2B and 2K). Activity in later task epochs was often tuned to nonvisual task variables such as choice side and outcome. For example, we observed units that preferentially fired during the movement epoch to one choice side over the other, and units whose activity during the outcome period was modulated by reward delivery (Fig 2F and 2G). Applying the selectivity index analysis to the choice epoch, we found that 47% (165/348) of single units that fired >1 spike/s in this epoch had choice side selectivity across all difficult trials, and thus had “robust choice selectivity” (Fig 2I), while 72% (250/348) were significantly side selective compared to shuffled data controls on at least 1 trial condition. We refer to this feature as “choice” throughout, though we recognize that it can be confounded with movement signals in our task, where choice reports are made by port-to-port movements. Movement-associated responses could be due to either the movement itself or movement-induced optic flow. Despite the low light levels in the behavioral arena, both of these remain possibilities. However, at the end of this section, we will analyze the relationship between “choice” and “movement” by comparing between port movements in different task epochs and will show that the choice epoch side-selective responses cannot be explained by movement-associated tuning. During the outcome epoch, 66% (200/306) had reward outcome selectivity (Fig 2J). Choice tuning was also significant in a sizeable proportion of units during the outcome epoch (42%, 127/306). Many neurons were selective for combinations of these 3 features across epochs (Fig 2L). Thus, choice and outcome strongly modulated single neuron activity in V1 during later task epochs, during which many neurons had their peak activity.

**Fig 3:**
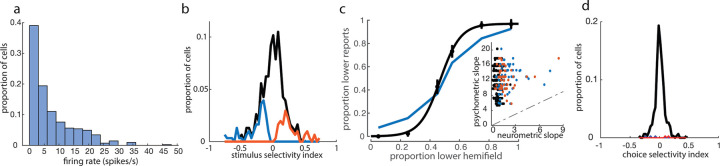
Stimulus epoch activity elicited by “cloud of dots” stimulus is spatially tuned, but less accurate than the animal’s behavior. (a) Firing rate distribution across putative single neurons during stimulus epoch. (b) Distribution of stimulus selectivity index across all cells active in the stimulus epoch. Blue (lower-preferring, 64/305) and orange (upper-preferring, 54/305) histograms denote cells with significant stimulus selectivity, compared to a shuffle control. (c) Comparison of psychometric (black) with neurometric (blue) curve for best lower-preferring cell. Inset: Comparison of psychometric and neurometric slopes across all single units used for stimulus selectivity analysis. Dashed line indicates unity line. (d) Selectivity index-based choice probabilities in V1 single neurons (see Methods). Cells with significant choice probabilities are shown in blue (3/305) and orange (2/305). The underlying data for this figure are available for download from 10.17632/5ms7gcb67j.1.

We then asked how the specificity of the stimulus-evoked neuronal responses compared to the animals’ behavior. Across the population, the firing rates during the stimulus period were typically modest (mean 7.2 +/− 7.8 spikes/s, median 4.7 spikes/s, Fig 3A), and only a minority (39%) of neurons that were active (>1 spike/s) during the stimulus presentation were selective for upper versus lower stimuli. Of those that were selective, most were weakly selective: Only about 1% of neurons (4/305) had a selectivity index greater than +/− 0.7 (Fig 3B). Across the population, no single unit matched the sensitivity of the animal’s performance on the corresponding session (Fig 3C). We also assessed the trial-to-trial variability in stimulus epoch firing predicting the animal’s choice, by using either a selectivity index or ROC analysis to estimate choice probability. Consistent with previous reports in primates [29], choice probabilities, calculated as the selectivity for future choice from stimulus period activity for a given stimulus condition, were low in V1, with only 2% (5/305) of cells having significant choice probabilities during presentations of difficult stimuli, relative to a shuffle control (Fig 3D). Choice probability calculated using ROC analysis produced similar results (7/305, S2A Fig). Thus, activity during the stimulus period reflected the true stimulus more than the perceived stimulus or upcoming choice.

**Fig 4:**
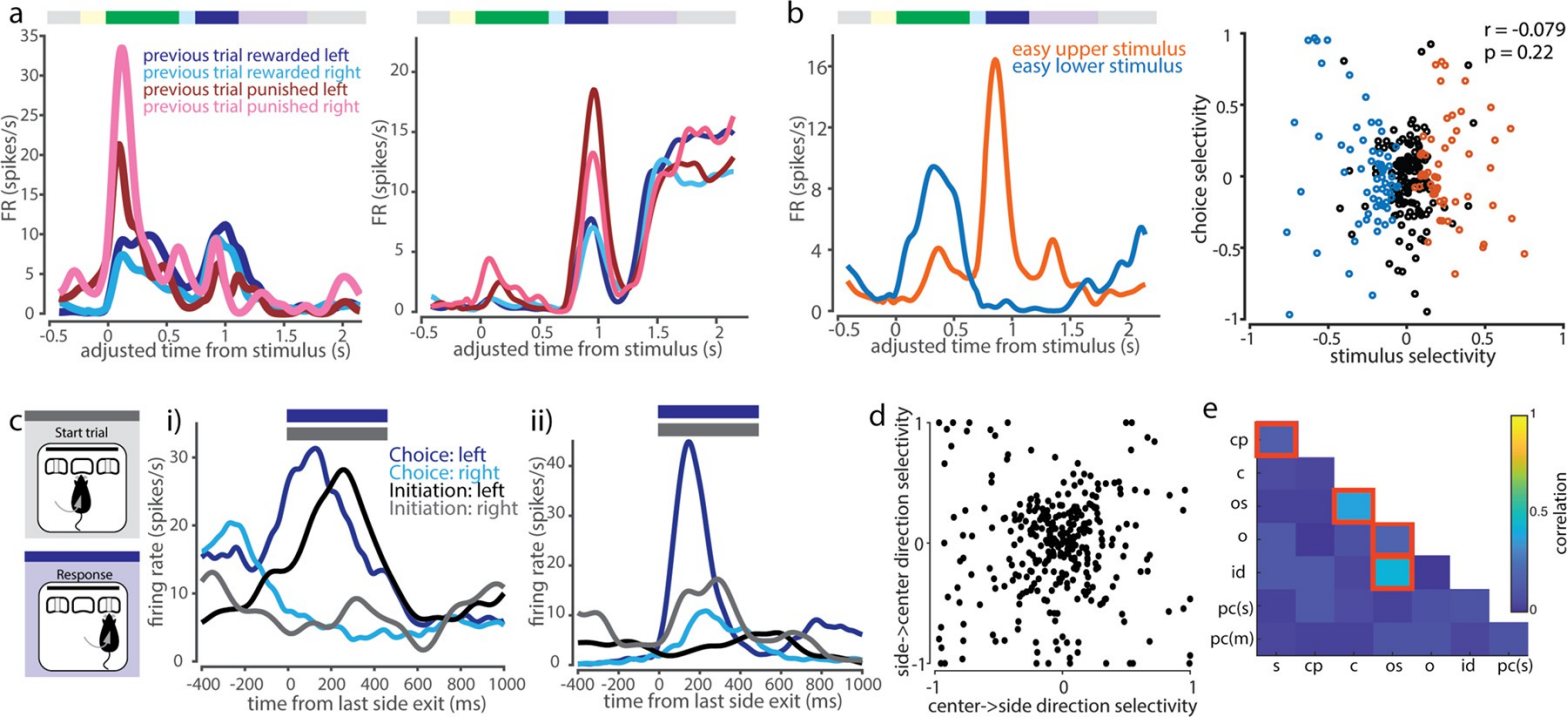
V1 single neuron tuning to non-sensory task variables. (a) Example cells showing modulation of task-related activity by previous trial behavioral variables during stimulus and/or choice epochs. (b) Left: Example cell showing anti-coherent tuning between stimulus and choice epoch. Right: No significant correlation between stimulus and choice selectivity across cells. (c) Comparison of between-port movement responses within movement-responsive cells (initiation epoch, gray, versus choice epoch, blue). (i) Example cell with similar leftward-preference during both task epochs. (ii) Example cell with varying side preference and amplitude of movement side-selective responses between initiation and choice epochs. (d) Side-selectivity index of between-port movements is uncorrelated between choice and initiation movements (Pearson correlation, r = 0.011, *p* = 0. 838). (e) Selectivity indices across pairs of task features are mostly uncorrelated within neurons. Highlighted squares indicate pairs of features that are significantly correlated (*p* < 0.05, Bonferroni corrected for multiple comparisons). Legend: s = stimulus, cp = choice probability, c = choice, os = outcome side, o = outcome, id = initiation direction, pc(s) = previous choice (stimulus period), pc(m) = previous choice (movement period). The underlying data for this figure are available for download from 10.17632/5ms7gcb67j.1.

To further understand non-sensory drivers of activity in V1, we asked whether non-sensory tuning was purely transient, arising only at the moment of the non-sensory event, or whether non-sensory task parameters could exert a persistent influence that spanned trials. We found that some cells were modulated by previous trial parameters, such as whether the previous trial was rewarded, and which choice port was selected in the previous trial (Fig 4A). Applying the selectivity index analysis as above to pre-stimulus baseline epoch activity, we found that 142/303 of active single neurons (47%) were selective for previous choice and 106/303 (35%) were selective for previous reward (S2E and S2F Fig). Such response profiles indicated that choice and outcome tuning do not only influence V1 activity transiently and instantaneously, but rather can be represented in a sustained or history-dependent manner within single cells.

We then asked if there is a systematic relationship between stimulus preference and choice preference in single units. About a fifth (21%, 51/239) of units tuned to either stimulus or choice were tuned to both. However, co-tuning could not be predicted from task contingencies, with tuning opposite to the reinforced association in about half of these neurons (47%, 24/51; Fig 4B). Across the population, we found no correlation between stimulus and choice side selectivity indices (Pearson correlation, *p* = 0.22). Thus, single neurons encoded combinations of stimulus and choice, including combinations that differed from task contingencies reinforced during training.

Similarly, we compared the responses elicited during the 2 between-port movements in our task: the center-to-side choice movement versus the side-to-center trial initiation movement. We found both cells that displayed similar tuning preferences and response dynamics across the 2 movements and cells that had different response amplitudes or tuning preferences (Fig 4C). For this analysis, we restricted initiation movements to those that were completed in <0.5 s between side port exit and center port entry, corresponding to direct port-to-port movements of similar latency as choice movements. There was no significant correlation across the population between tuning direction and magnitude, when calculated by selectivity index, across these 2 epochs (Fig 4D). Thus, movement-direction tuning appeared to be modulated by task epoch.

We repeated this correlation analysis for all pairs of task variables using the selectivity measure described above (Eq 1). There was in general no systematic relationship between tuning preferences: we observed predominantly weak, insignificant correlations between selectivity to most pairwise combinations of task variables, indicating that tuning preferences were largely independent across task features (Fig 4E).

Taken together, these analyses show that responses in V1 during this task are driven by features not only limited to sensory input, but also including movement direction and outcome, sometimes influenced by multiple parameters, such as previous trial features or current task epoch.

### V1 neurons encode diverse, unstructured combinations of stimulus and task variables within and across task epochs

**Fig 5:**
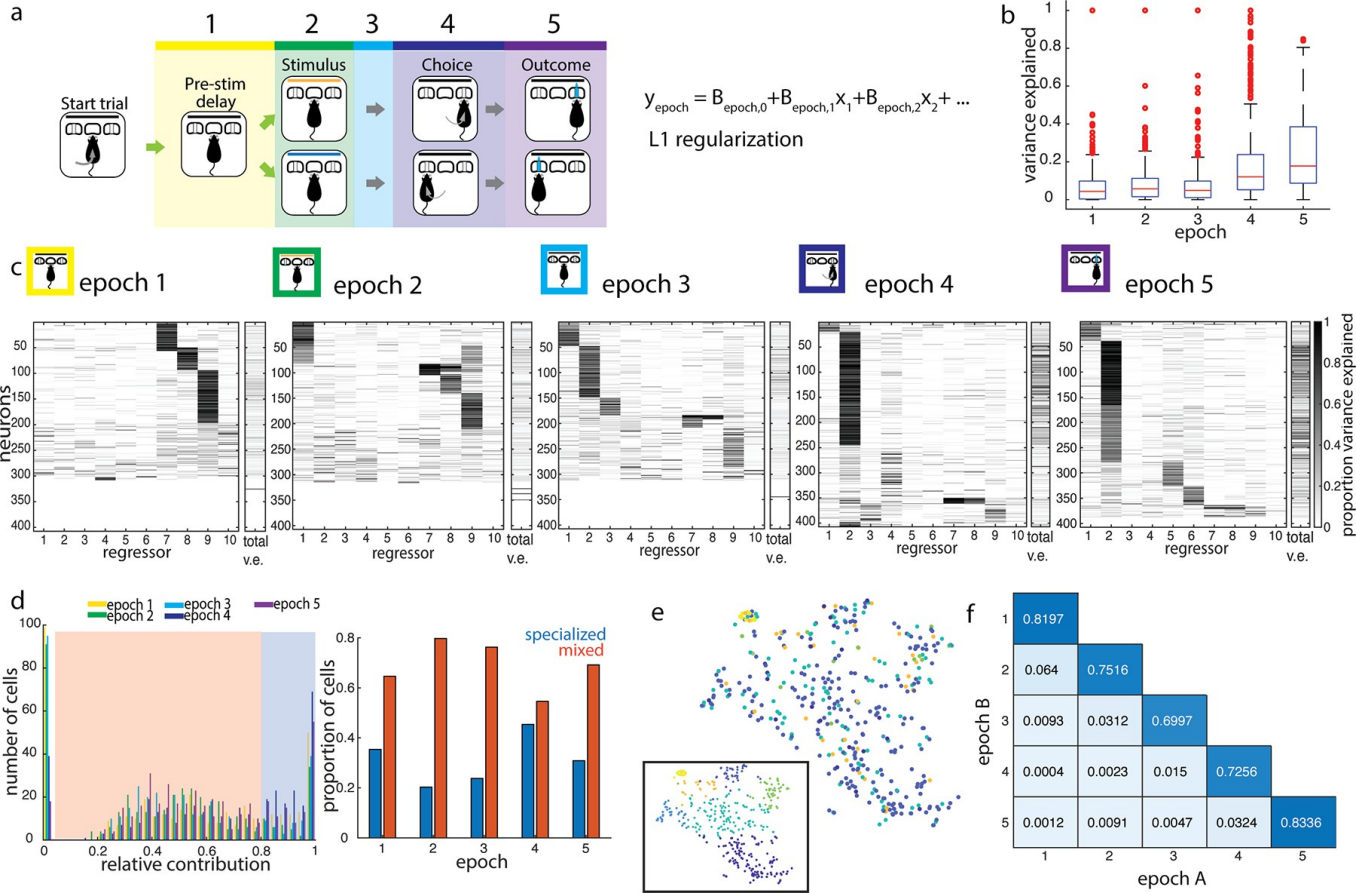
Single neurons represent combinations of task features within and across task epochs. (a) Design of linear encoding model. Trial divided into 5 epochs, as marked. Linear model was fit using 10 task parameters to predict trial-by-trial firing rates within epochs: (1) stimulus, (2) choice, (3) reaction time, (4) movement latency, (5) correctness, (6) reward delivery, (7) previous trial last port visited, (8) previous trial choice, (9) previous trial outcome, and (10) previous trial stimulus. (b) Box and whisker plot of total variance explained by the model, by epoch. (c) Relative variance explained by individual regressors in the linear encoding model, by epoch. Total variance explained for each neuron is shown in the rightmost column in each epoch. The left 10 columns show the proportion of the explainable variance attributed to each regressor for each neuron (darker shading = higher proportion of total variance explained, see Methods). Neurons (rows) are clustered and sorted within epochs. In some units, single regressors dominate the explainable variance, while in others, multiple regressors contribute to the encoding model, revealing the presence of both “specialized” and “mixed” encoding by cells during each epoch. (d) Distribution of maximum contribution by a single task parameter to predictions. Thresholding at a relative contribution of 0.8 separates cells into “mixed” (orange shading) and “specialized” (blue shading) encoding profiles. Cells with maximum relative contribution near 0 are excluded as not being well-driven by any of the regressors. Right: Proportions of specialized versus mixed encoding cells across epochs. (e) t-SNE embedding of encoding profiles of single units in the outcome epoch, clustered by cluster identities from the choice epoch. Inset shows the same embedding, clustered by outcome epoch cluster identities. Color denotes cluster identity. (f) Cluster goodness-of-fit measure (adjusted Rand Index; see Methods) for all pairwise comparisons of epochs A and B. Clustering different epochs produces fewer shared cluster members than 2 independent partitions of the same epoch. The underlying data for this figure are available for download from 10.17632/5ms7gcb67j.1.

Having observed a variety of single neuron response patterns in V1, we next set out to quantify the relative influence of different task variables on single neuron activity over the course of a trial. To systematically interrogate how task features influenced single neuron activity at different points in the task, we fit a linear encoding model to estimate the relative influence of each task feature on the firing rate *y* of a given neuron during task epoch *i* (Fig 5B),

$$y_{i}=\beta_{i,0}+\beta_{i,1}x_{1}+\beta_{i,2}x_{2}+\cdots +\beta_{i,10}x_{10}$$ (2)

where *i* = 1…5 denotes the task epoch; *x*_1_…*x*_10_ denote the following behavioral variables: stimulus identity (*x*_1_), choice (*x*_2_), reaction time (*x*_3_), movement latency (*x*_4_), choice correctness (*x*_5_), reward delivery (*x*_6_), port last exited on the previous trial (i.e., port visited directly preceding initiation poke, *x*_7_), previous trial choice (i.e., port first visited at previous trial decision time, *x*_8_), previous trial outcome (*x*_9_), and previous trial stimulus identity (*x*_10_); *β*_*i*,1_…*β*_*i*,10_ are their corresponding weight coefficients within epoch *i*, and *β*_*i*,0_ is the intercept. Note that behavioral variables do not depend on the epoch, as each takes on only 1 value per trial, i.e., each trial has only 1 choice side, 1 reaction time, etc. The model was fit using Lasso regularization with 10-fold cross validation, to derive weights to identify the most informative behavioral variables. We quantified the total variance explained by the model, as well as the relative contribution of each of those variables, by comparing the variance explained by the model when including versus excluding each variable.

In previous analyses above (Fig 2), we observed that a larger fraction of single neurons in V1 responded during choice and outcome epochs than during the stimulus presentation. Consistent with this, we found that the model also explained a larger total proportion of the variance of choice and outcome epoch activity (mean variance explained of 0.19 and 0.25, respectively, Fig 5B), compared to the stimulus epoch (mean variance explained of 0.09; distributions are significantly different by the Kolmogorov–Smirnov test, *p* < 10^−14^ for both). While the variance explained by this linear model may be considered a lower bound, and we expect that a model including more complex response functions and more regressors related to body posture would likely perform better, this model captured several properties of V1 single neuron encoding. Choice again emerged as a prominent feature encoded throughout much of the trial, beginning in epoch 3, prior to the onset of movement. Furthermore, within the stimulus epoch, we found more total cells whose activity was better explained by one of several previous trial task features, such as previous choice, outcome, and exit port side, than by current stimulus identity. Features encoded by single neurons therefore transitioned from previous trial choice and outcome in early task epochs to current trial stimulus to current trial choice and outcome in late epochs, with stimulus representations explaining a minority of responses in any epoch. Thus, single neuron firing variability was consistently better explained by non-stimulus task variables, over the course of the trial and even during stimulus presentation.

Of the activity explainable by our model, we wanted to know whether cells were predominantly “specialized” for encoding a single task variable or encoded a “mixture” of task variables. Based on the distribution of the most prominent task feature’s contribution to the linear model, we set a cutoff that classified features surpassing a relative contribution of 0.8 as dominating a given neuron’s response and that neuron was subsequently designated as “specialized” during that epoch. Otherwise, the neuron was designated as having “mixed” representations, with more than 1 task variable contributing substantially to its activity in that epoch. In most epochs, the majority of single neurons (between 55% and 80%) were driven by a combination of task features, rather than a single feature. The closest ratio was in the choice epoch, where there were almost as many specialized choice-selective neurons as there were neurons encoding a mixture of stimulus, choice, and other movement-related features such as reaction time (Fig 5D). Therefore, task information was encoded not by multiple independent groups of specialized cells, but rather by overlapping modulation of the activity of single cells.

The predominantly mixed profiles of neural responses argue against a simple labeled line model, in which each task variable is represented by a particular class of cells receiving input predominantly from a single source. We therefore considered a somewhat more complex model in which neurons within a cell class represent similar sensory and non-sensory variables between them, across epochs, i.e., 2 neurons that represent the same combination of features in the stimulus epoch will also look similar to one another in their encoding patterns in the choice epoch. To test this, we clustered neurons on the basis of the relative contributions of all task features in a given epoch (e.g., choice epoch), and used these clusters to sort the relative contribution of task features to their activity in each of the other epochs (e.g., outcome epoch, Fig 5E). We found that no distinct clusters emerged in the outcome epoch, when cells were ordered by their cluster identity in the choice epoch. We repeated this for all clustering epoch–test epoch pairs and saw that cluster identity always generalized poorly across all pairs of epochs (Fig 5F). This is reflected in the adjusted Rand Index, a standard measure that quantifies the overlap in cluster membership between 2 independent partitions and was much lower for cross-epoch comparisons than within-epoch comparisons. The adjusted Rand Index, which ranges between 0 and 1, is maximized when the same sets of neurons are clustered together in both partitions. Thus, single neurons represent diverse combinations of task variables both within and across epochs, without any evident organization or structure.

### Current and past trial task features can be decoded from V1 population activity

**Fig 6:**
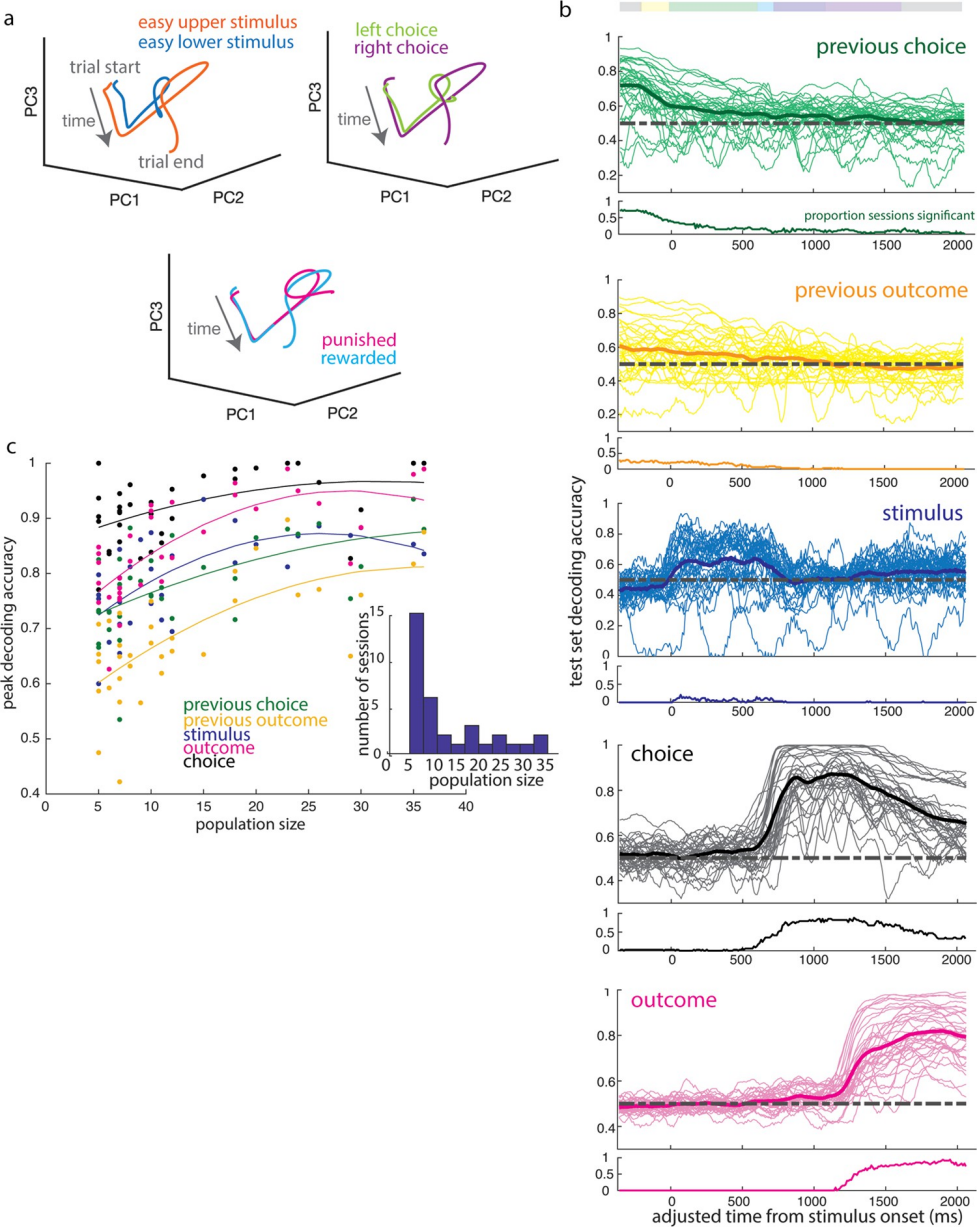
Reliable decoding of task variables across trial duration from trial-by-trial population activity. (a) Mean population activity trajectories for an example session, projected onto the first 3 principal components, separate over the course of the trial by stimulus, choice side, and outcome. (b) Decoding accuracy in sliding 100 ms bins over the course of a trial for features of the previous trial (choice side, outcome) and of the current trial (stimulus category, choice side, and outcome). Thin lines correspond to individual sessions, while bold lines denote the mean across sessions. Decoding accuracy is calculated as proportion of test set classified correctly from activity at a given time point, and below each panel is shown the proportion of sessions in which decoding was significantly more accurate than when labels were shuffled. (c) Maximum decoding accuracy of trial features as a function of population size. Fitted lines correspond to the best fit second-order polynomial function, shown for visualization purposes. Dependence of decoding accuracy on population size was confirmed using Spearman’s rank correlation coefficient (previous choice: rho 0.5994, *p*-value 1.79e-4; previous outcome: rho 0.6466, *p*-value 3.61e-5; stimulus: rho 0.6558, *p*-value 2.6e-5; choice: rho 0.4481, *p*-value 7.9e-3; outcome: rho 0.7307, *p*-value 9.27e-7). Inset: Distribution of population sizes. The underlying data for this figure are available for download from 10.17632/5ms7gcb67j.1.

The single neuron encoding patterns we observed suggested that the encoding of task variables was distributed across a heterogeneous V1 population. Such shifting representations at the single-cell level may nonetheless underlie stable representations at the population level. We therefore analyzed the information available in populations of simultaneously recorded cells throughout the duration of a trial. First, we used dimensionality reduction methods to inspect the population activity of simultaneously recorded units (both putative single units and multiunit activity) over the course of single trials (Fig 6A). Activity patterns diverged over the course of the trial on the basis of stimulus identity, choice side, and outcome, and evolved along distinct dimensions during the stimulus, choice, and outcome periods. This suggested that it would be possible to read out these task features from V1 population activity at different points in the trial.

To test how well features of the task could be decoded from the population activity at each time point, we trained a linear classifier to decode task variables: stimulus category, choice, and outcome, previous choice and previous outcome (Fig 6B). We found characteristic decoding timecourses for each feature. Stimulus category could be decoded primarily during stimulus presentation (see Methods: Decoding (Linear Classifier)). Task features associated with the previous trial, such as previous choice and previous outcome, could be decoded early in the trial, with performance decreasing over the course of the trial. Consistent with this, choice and outcome were readily decoded both during and following their respective epochs. Outcome information could be decoded regardless of whether we pooled missed reward and punishment outcomes or treated them separately (S3C Fig). The timecourse of how well each feature could be decoded from the neural activity was similar across sessions for any given feature, which is reflected in the proportion of sessions with significantly better-than-chance decoding accuracy over the course of the trial (Fig 6B). Thus, multiple task features could be read out from population activity at each time point over the trial, including during early epochs when single neuron activity was less well explained by the previous encoding model.

Decoding accuracy improved on sessions with more simultaneously recorded units, but notably, even the smallest populations included in this analysis (5 units) were able to exceed a decoding accuracy of 60% for most task features (Fig 6C). In addition, classifier performance did not increase substantially with population size beyond about 20 units. Thus, despite the heterogeneity of single neuron activity patterns, task information could readily be decoded by a linear decoder from small V1 populations, with a similar timecourse over sessions.

### V1 representations during visually independent choice task

**Fig 7:**
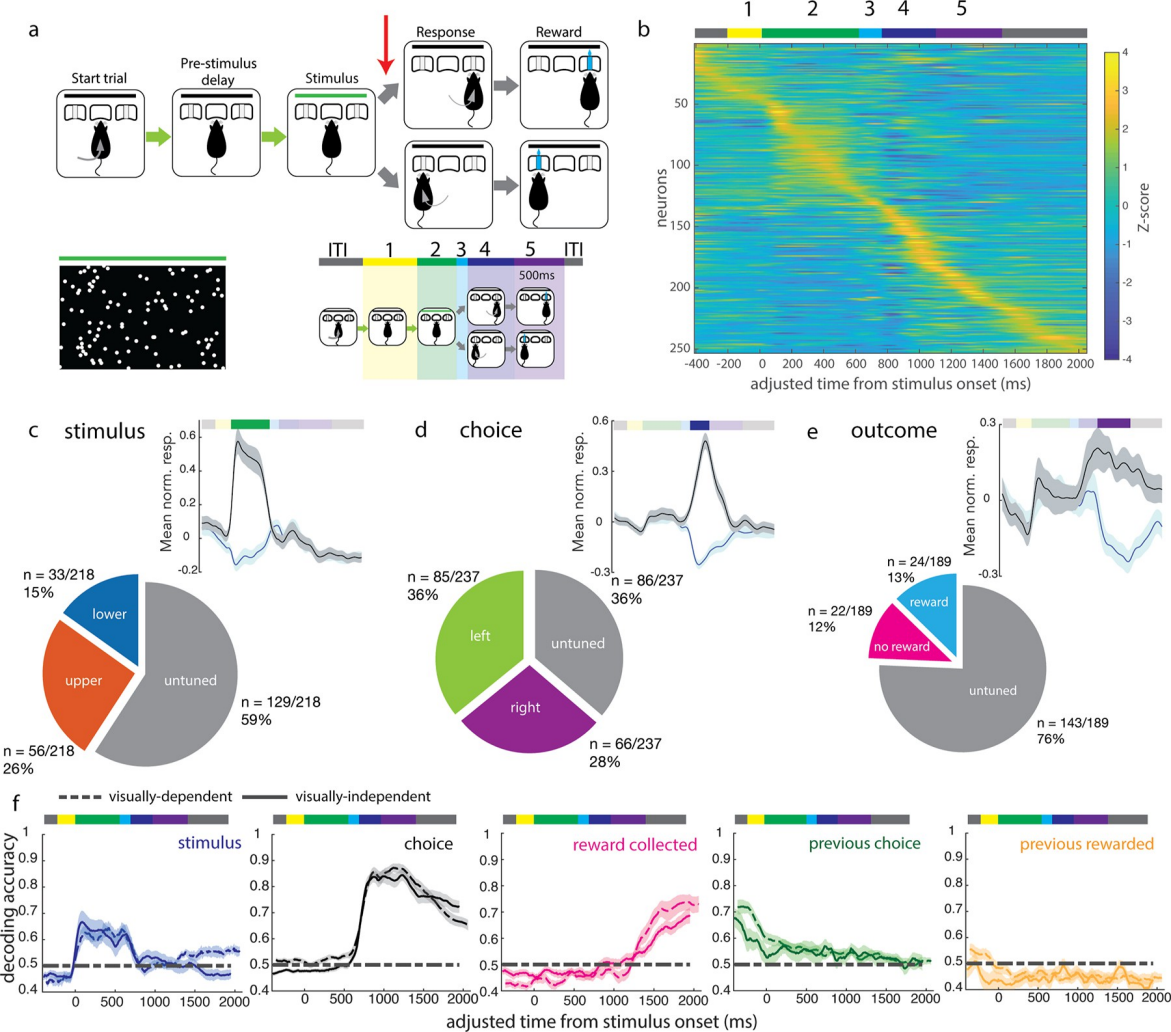
V1 responses during a visually independent decision task. (a) Task structure is identical to the structure of the visual decision task, except that decision tone (red arrow) is presented on 1 side only. A response to the same side as the decision tone yields a reward. (b) Z-scored mean activity of single units, sorted by time of peak activity. (c–e) Normalized mean response (mean z-scored activity and SEM) of selective single units for (c) stimulus, (d) choice side, and (e) reward delivery, with black denoting response to the preferred feature, and blue denoting response to the non-preferred feature, and the proportion of the population with selectivity for each feature. (f) Mean decoding trajectories over visually independent decision sessions (solid lines) for current trial stimulus, choice, and outcome, previous trial choice, and previous trial outcome. Dashed lines denote mean trajectories during the visually guided decision task, as shown in Fig 6, and shaded areas denote SEM. The underlying data for this figure are available for download from 10.17632/5ms7gcb67j.1.

The robust task-related representations we observed in V1 could be specific to visually guided decisions. Alternatively, non-sensory representations might be encoded in visual cortex independently of whether primary visual cortex is required for the decision process. To distinguish these possibilities, we interrogated V1 responses in a new cohort of subjects trained to perform a similarly structured task in which decisions were based on auditory rather than visual stimuli. In this modified task, visual stimuli were presented but not informative for the animal’s choice. Instead, animals were instructed as to the correct choice based on the location of the decision tone, which was presented on the side of the animal corresponding to the correct side port for that trial. The task structure was otherwise identical to that of the visual decision task (Fig 7A). During this task, the visual stimuli consisted of randomly dispersed dots over the full extent of the monitor on the majority (70%) of trials. On the remaining trials, animals were presented with one of the 2 “easy” stimuli from the visually guided decision task. Animals acquired this task to near perfection, and their choice profiles were uncorrelated with the distribution of the visual stimulus (S4 Fig).

We recorded from 253 well-isolated single units and 41 multiunits from 2 animals performing this task variant. The trial-averaged activity across the population was similar to that recorded in the visual decision task, with the majority of units having their peak firing during or after the movement epoch (Fig 7B). Firing rates were similarly modest, with a mean of 6.5 (+/− 4.4 std) spikes/s (S6A Fig). Stimulus selectivity profiles were also similar between the 2 tasks: 41% of single units were stimulus selective in the visually independent task (Fig 7C). The proportion of choice selective cells increased from the proportion of robust choice selective cells in the visually guided task (64% compared to 47%, Fig 7D), while the proportion of outcome selective cells decreased. Because errors were rare in this task, we instead compared rewarded versus missed reward trials (i.e., a correct choice where the animal’s choice port nosepoke was too short in duration to trigger a reward); 35% versus 24% of cells in the visually guided and visually independent tasks were selective between rewarded versus missed reward trials, respectively (Fig 7E). In the pre-stimulus period, 29% of cells recorded in the visually independent task were selective for previous trial choice (S5C Fig), compared to 42% in the visually guided task. Meanwhile, 14% was selective for whether the animal collected versus missed the reward on the previous trial in the visually independent task (S5D Fig), compared to 12% in the visually guided task.

Decoding task features from population activity yielded timecourses similar to those obtained on the visually guided task, with some differences in peak decoding accuracy. While stimulus and choice decoding were accurate to similar levels as in the visual decision task, the decoding performance for rewarded versus missed reward trials showed a trend toward decreasing in the visually independent task. This effect was weakly significant when tested by the Mann–Whitney U test (*p* = 0.0032) in which sessions from all animals in each task were grouped together, as is often done in systems neuroscience. However, when tested using hierarchical statistics [30] which take into account the possibility of batch effects caused by correlations within each animal, the effect was not significant (Figs 7F, S7A–S7C and S7F). In addition, the onset of significant outcome decoding was delayed to after the first 500 ms of the outcome epoch (Figs 6B and S7C). The slower and decreased rise in outcome information is consistent with the execution of different motor programs following reward versus no reward, in the late outcome period. Previous trial choice and outcome decoding accuracy were also reduced during the visually independent task (Figs 7F and S7D–S7F). These effects were weakly significant when sessions from all animals were grouped together (Mann–Whitney U test, *p* = 0.0475 and *p* = 0.0452), but not significant when tested using hierarchical statistical methods as above (see Methods).

Finally, when fitting the same linear encoding model across the 2 tasks, we found that single neuron activity in the visually independent decision task was (1) similarly predominantly driven by more than 1 task feature at a time; and (2) similarly better described at later points in the trial (choice and outcome epochs) than at early points in the trial (including the stimulus epoch, S6B–S6D Fig), as in the visual decision task (Fig 5). There was a trend toward a decrease in the total proportion of the variance explained by our model in each epoch in the visually independent decision task, compared to the same epoch in the visual decision task (S6E Fig). Similar to the results of the decoding analysis (S7 Fig), these comparisons were significant when neurons from all animals within each task were grouped together (Mann–Whitney U test, *p* < 0.005 for all comparisons) but were not significant when tested using hierarchical statistical methods that account for batch effects (see Methods). Taken together, these results suggest but do not establish rigorously that there is a decreased influence of some task features on V1 activity during the visually independent task.

The comparison of the visually guided task with the nonvisual task suggests that while neural activity in V1 was broadly similar between the 2 tasks, encoding of the non-sensory task features we investigated here—choice and outcome—was weakly modulated by the behavioral context. Representations of outcome in single cells and across the population were less prominent in V1 during visually independent decisions, while representations of choice remained robust. Previous trial features were also less well represented at the population level, further suggesting that processing of non-sensory information in V1 in a freely moving animal depends somewhat—but not entirely—on the behavioral demands related to visual processing.

## Discussion

In this study, we developed a novel visual decision task for freely moving rats to study representations in primary visual cortex during freely moving visual decisions. By recording single unit activity during this behavior, we found robust tuning for both sensory and non-sensory task features, and that tuning preferences were distributed and independent of stimulus-choice contingencies. Single cells were more likely to be driven by multiple features in each epoch than a single task feature. Task features could be decoded from small simultaneously recorded populations of units, with previous trial features best decoded early in the trial, and giving way to current trial features as the trial progressed. Finally, many of the tuning patterns described for the visual decision task held true during a visually independent variant of the task, with some modulation of the representations of outcome and previous trial task parameters.

This study complements and adds to a growing literature on visual cortex responses during decision-making in head fixed mice. Here, we removed the motor constraints that are placed on head fixed mice and found that nonvisual task variables remain prominently represented in visual cortex. To perform these experiments, we developed a virtual head fixation protocol that is noninvasive, compatible with experimental techniques, and learnable without a direct reinforcement signal. This allowed us to restrict the viewing angle of visual stimuli in a freely moving animal, which we combined with well-defined choice reports and measures of behavioral timing. This system allowed us to impose a real-time postural criterion into training protocols for our task. At the time these experiments were initiated, deep-learning–based pose estimation algorithms were not yet available for implementation of real-time video tracking and reactive control of behavioral hardware [31], although they have since been developed [32,33] and could be used to refine this training approach.

The presence and organization of task representations in visual cortex have implications for the computations that can occur locally and in circuits involving V1. In frontal and parietal cortices, where representations of diverse task-related variables are more frequently studied, there is debate as to whether representations are randomly assorted across neurons, or organized into discrete classes, with potential implications for downstream decoding [34]. Recent work has identified distributed encoding profiles in both cortical [35] and subcortical brain regions. In VTA dopaminergic neurons, different degrees of specialization arise in different task epochs [36], and the specific variables encoded by a given neuron also varies across task epochs. Here, we observed similar complexity in the encoding patterns in a primary sensory cortical area, V1, with cells tuned to the same variable during one task epoch later representing different variables between them in a later epoch, with uncorrelated tuning preferences. Within individual epochs, representations of a given task feature were distributed across the population. In the stimulus epoch, single neurons were less accurate than the animal at classifying the incoming stimuli, and over the trial, both sensory and non-sensory task parameters were decoded better with increasing neural population size of up to approximately 20 units. Taken together, our results suggest that the primary visual cortex may share some organizational principles with frontal and parietal areas, in that task feature representations are distributed across neurons.

In agreement with recent literature in head fixed mice, we find prominent non-sensory representations in V1, with a greater proportion of neurons identified as selective for non-sensory features such as choice and outcome than were selective for the visual stimulus. There are 2 characteristics of our dataset that arise from the design of the task. First, we presented stimuli that were designed to provide a distributed visual signal, rather than maximally drive individual V1 neurons. While we believe this is more indicative of the normal operating regime of the visual system, because of this, visually driven responses were likely weaker than would have been observed with other stimuli. Additionally, because we determined visual selectivity as tuning between the upper- and lower-dominated visual stimuli, our estimate of the fraction of visually selective neurons may represent a lower bound on the true number of visually responsive neurons sampled, as some visual neurons may have more complex tuning than we were able to probe using our stimulus design. Second, while responses of V1 neurons have previously been found to be modulated by head movements [17,37,38], in our task, such movements are highly correlated with task parameters such as categorical choice and related whole body movements. Because head movements are only permitted in our task while the visual stimulus is off (i.e., in the relative dark), previous work suggests that such movements would transiently suppress V1 activity. While we find that most neurons have their peak firing during such dark epochs of the task, this activity is likely due to the sum of effects of whole body and head orienting movements, which we choose to summarize in relation to task parameters (e.g., choice or reward collection).

One striking observation was that the ability to decode task features from V1 populations could extend well past the event’s duration, into the next trial, during visually guided but less so in visually independent decisions. This argues against the possibility that non-sensory responses in V1 merely reflect an instantaneous “echo” of a brief event such as a motor command. Rather, visual cortex has the ability to carry sustained representations of different task parameters, with possible modulation by task demands (Figs 7 and S6 and S7). Recent work has suggested that non-sensory responses in V1 help shape sensory processing by influencing the correlation structure and population activity space [39]. Here, we found that sensory processing requirements may influence the strength of non-sensory representations in V1.

Which characteristics of the task might modulate the strength of non-sensory representations in V1? Because our 2 tasks are identical in trial structure, but differ in whether the animal is required to use a visual stimulus to guide its behavior, we suspect that a possible relevant characteristic is whether the task requires visual processing. Another possibility is that V1 task representations depend on the overall difficulty level of the task, i.e., whether difficult (perceptually ambiguous) trials are included. Future work could seek to further characterize changes in V1 activity along both of these task dimensions by investigating V1 activity during perceptually difficult auditory-guided decisions. Either way, flexible routing of task-related information through V1 would suggest that non-sensory representations may serve a task-dependent computational role. For example, previous-trial parameters may support learning of expectations about the structure of the task and stimulus space.

The stimulus-choice associations that animals were trained on were not reflected in the co-tuning preferences of single cells (Fig 4). This was surprising in light of previous studies [22,40], in which coherence between visual encoding and behavioral response emerged over training. There are a number of differences in these tasks that could account for these differences. First, in previous studies, the visual stimulus and the appropriate response overlapped in time, whereas in our task they were temporally separated. Second, in previous studies, the stimuli and eventual outcome were deterministically paired (e.g., only 1 stimulus could lead to reward), whereas in our task both stimulus categories were equally likely to lead to reward. Finally, there are differences in the V1 neuronal populations sampled: the previous work used 2 photon imaging, which predominantly samples neurons in layer 2/3, whereas in our study, we used tetrodes and thus sampled deep layers as well. Layer 5 neurons in V1 tend to have larger and more complex-like receptive fields (e.g., wider orientation tuning curves, [41]), and it has been hypothesized that layer 5 V1 neurons may carry out distinct computational functions compared to neurons in layer 2/3 [42]. Future work delineating the behavioral limits where coherence between sensory and non-sensory representations no longer develops may provide clues to how visual cortex processes non-sensory information to support different tasks.

In the context of recent work, our study adds to the growing evidence that the range of responses measured in visual cortex extends far beyond visual stimulus-driven activity. In particular, we contribute evidence for diverse, distributed task representations in V1 in freely moving rodents, complementing the growing literature on V1 activity in awake head-fixed rodents.

## Materials and methods

### Ethics statement

All procedures were approved by the Cold Spring Harbor Laboratory Institutional Animal Care and Use Committee (approval number 22-19-16-13-10-07-03-00-4) and conducted in accordance with the National Institutes of Health guidelines.

### Animals and surgical procedures

Approximately 8- to 10-week-old male Long Evans rats were obtained from Taconic Biosciences and Charles River and started training after reaching at least 10 weeks of age. Rats were pair-housed until implantation of the microdrive, after which they were singly housed, in a reverse 12 h light/dark cycle. Implant surgeries were performed under 2% isoflurane anesthesia. Custom-built microdrives were implanted according to stereotaxic coordinates, with the tetrode bundle targeted to left binocular primary visual cortex (bregma– 6.1 mm AP, +4.5 mm ML).

### Task design and behavioral system

Custom behavioral chambers consisted of 3 ports attached to a clear wall panel through which a monitor was visible to the interior of the behavioral box. Interruption of an infrared beam inside the ports was used to determine timing of port entry and exit. We used the Bpod system (Sanworks, NY) to implement the behavioral state machine. The task structure was as follows: animal entry into the center port triggered the beginning of a pre-stimulus delay. The variable pre-stimulus delay was drawn from an exponential function with a mean of 0.3 s. Following this delay, a 500 ms fixed time stimulus was delivered through Psychtoolbox [43–45]. A 200 ms fixed poststimulus delay separated the stimulus off trigger from the decision tone. Any withdrawal from the center nosepoke at any point between the pre-stimulus delay initiation and the decision tone delivery led to a missed trial and a 2 s time out. After implementation of the head position protocol, a missed trial could also be triggered by a head movement while in the center port during this peristimulus period. After the decision tone, the animal was given 3 s to make a decision by poking into a side port. A 20 μl reward was delivered following a 50 ms nosepoke into the correct port. A correct choice report that did not fulfill this duration requirement did not trigger reward, but no punishment was delivered either. No intertrial interval was specified following correct (either rewarded or missed reward) trials. A 1 s punishment tone (white noise stimulus) and a 5 to 6 s time out followed an incorrect choice.

The Psychtoolbox toolbox was used to generate and deliver visual stimuli and auditory decision and punishment tones. For each stimulus, 30 frames were delivered at 60 Hz refresh rate, with stimuli randomly distributed across each frame according to the stimulus condition on that given trial. For the visually guided decision task, the stimulus consisted of 2 subregions of equal size, separated by a thin boundary region where no dots were ever present. From the position of the animal, the lower subregion subtended 0 to 38 degrees of elevation in the animal’s visual field and the upper subregion 42 to 60 degrees of elevation. Horizontally, the stimulus subtended 94 degrees along the azimuth, centered on the animal. For the visually independent task, dots were presented across the full extent of the display. For the visual decision task, the less dense subregion on each frame was given the number of dots drawn from a Poisson distribution centered on the lesser mean dot value of that stimulus condition. The denser subregion was given the complementary number of points. Therefore, every frame had the same total number of individual dots. Each dot location corresponded to a round white dot that subtended about 3° in visual space. Dot locations were drawn from a uniform grid where every tenth pixel was a possible centroid. Of these possible locations, only 1% was selected as active on any given frame. Because dots were sparse, but dot size exceeded the spacing of the grid (30 pixel diameter), overlap was possible but minimal. Maximum overlap occurred on “easy” trials where 95% of dots appeared in a single subregion. In these trials overlap was on average 5% of the dot-occupied area and did not exceed 11%. A luminance detector module (Frame2TTL, Sanworks) reported luminance changes during each trial and the onset of stimulus delivery by detecting a reporter pixel which flickered on/off with each frame update.

### Head position control

We implemented the closed-loop head position condition using Bonsai, a reactive programming software [27]. Bonsai was given video input from a webcam (Logitech) mounted above the animal at a 70° angle. This video input was binarized and regions of interest (ROIs) were defined on a per-animal basis from this field of view. These ROIs were centered on the position of each ear, such that the ear would entirely fall within the ROI when properly aligned. At the level of the animal’s head, each pixel corresponded to 0.3 mm in real space. Built-in Bonsai functions carried out contour mapping of the image within each ROI and filtered viable objects on the basis of size. The centroid positions of the resulting objects were calculated, and if their distance did not exceed a threshold of 10 to 15 pixels, a binary signal representing the animal’s successful alignment was sent to the behavioral state machine. This condition was only tested for when the animal was in the port to prevent spurious detections or noise caused by background (e.g., behavior rig floor) objects. The algorithm performed a moment-to-moment “and” computation on the comparison between the x values, the comparison between the y values, and the input trigger to output a binary trigger back to Bpod. The continuation of the Bpod states depended on the continuous on-state of this trigger. To ward against fast software- or camera-generated errors from producing false negatives, a short 50 ms grace period followed every on-off transition of the trigger. If during this grace period, the trigger returned to the on state, the trial was allowed to continue; otherwise, it was aborted.

### Extracellular recordings

Tetrode drives were custom-built using Omnetics 36-pin EIBs and custom 3D printed drive skeletons. Each drive contained 8 tetrodes and 1 reference tetrode that traveled together in a single bundle. Subjects were implanted with tetrode drives under 2% isoflurane anesthesia following successful acquisition of both the visual decision rule (where applicable) and the head position requirement.

We used the Intan-based OpenEphys recording system to acquire neural signals. Four of the 7 animals reported here required light anesthesia to facilitate attachment of the recording tether (2/5 on the visually guided task and 2/2 on the visually independent choice task). These animals were given 15 min to fully recover before the task began. After each recording session, tetrodes were lowered by 40 to 80 μm. Recordings were made until tetrodes reached a depth of 1.5 mm. We electrolytically lesioned at the tetrode tips, after which animals were killed and brains were recovered for histology.

Spike times were extracted through semi-automated spike sorting using Kilosort software [46] on the raw continuous recording traces. The data was bandpass filtered and the mean across all channels was subtracted from all traces to remove any common noise events. We performed manual curation of detected spikes on the basis of their: amplitudes, waveforms, auto- and crosscorrelograms, firing dynamics over the session, and clustering in feature space. We further restricted single cell representation analyses to units with refractory period (2 ms) violations of less than 1%. All analyses were performed in Matlab.

### Time adjustment/neural data preprocessing

Individual trials varied slightly in duration due to variable durations of pre-stimulus delays, reaction times, and lengths of stay in reward ports. For all analyses that did not rely on mean epoch firing rates, to allow comparisons of firing rate trajectories over trials and sessions, e.g., in Figs 2, 6, and 7, we first “stretched” individual trials to a common timecourse across all recorded sessions. We sampled individual activity traces at regularly spaced time points within each epoch, and then mapped those sampled points back to the mean trial timecourse.

### Selectivity analyses

To find the selectivity of a cell’s firing during various task epochs, a selectivity index was calculated on the mean firing rates between pairs of trial types defined by the task parameter of interest. We defined selective cells as those whose selectivity index exceeds the 95% bounds of a shuffle control distribution. The shuffle control distribution for a given cell was built by calculating the selectivity index across 1,000 shuffles where the trial labels (e.g., upper or lower stimulus) were shuffled relative to the single trial firing rates for that cell. We carried out the same analysis to define movement side-selective cells during the choice epoch and reward-selective cells during the outcome epoch. For each epoch of interest, of the total single units (*n* = 407), only those with an average firing rate of more than 1 spike/s during that epoch were included in this analysis (stimulus epoch: 305 cells; choice epoch: 348 cells; outcome epoch: 306 cells; pre-stimulus baseline epoch: 303 cells).

Selectivity analyses in Figs 2–4 were calculated for variables including: stimulus (more upper dots versus more lower dots); choice (left port entry at decision tone versus right port entry); choice probability (eventual choice, neural activity during stimulus delivery); outcome (rewarded versus not rewarded); outcome side (left port during outcome epoch versus right port); initiation direction (approach to center port from left versus right port); and previous choice (left versus right port selected on previous trial).

### Neurometrics

ROC analysis was performed using the Matlab *perfcurve* function, using task variable as a binary label, and mean single trial firing rates in a given task epoch as the scores. To build the neurometric curve, we applied ROC analysis at each of the 3 stimulus difficulty levels presented, and took the area under the curve as the cell’s ability to discriminate between the 2 easy, the 2 medium, and the 2 difficult stimuli. These values were mirrored across the 50% point of the decision axis to estimate the full psychometric curve. For comparison of the slopes of the neurometric and associated psychometric curves, we fit a logistic function to the 6 points from the auROC analysis and a second logistic function to infer the psychometric function from the choice behavior and compared the slope parameter from these 2 fits.

### Linear encoding model

We trained a linear model to predict the firing rate during each epoch given the set of behavioral predictors. Binary variables (e.g., choice, correctness, and reward delivery) were coded as values of −1 and 1. Continuous-valued variables (e.g., reaction time and movement duration) were z-scored over the session. Stimulus identity took on a value between −1 and 1 which represented the comparison strength in the stimulus (proportion of dots_lower_−proportion of dots_upper_). We used Lasso regularization, setting lambda to minimize the deviance across validation sets. We carried out this model optimization using the Matlab *lassoglm* function, with 10× cross-validation. Variance explained by the model predictions (*η*^2^_*model*_) was used as a measure of model fit, calculated as:

$${\eta^{2}}_{model}=1-\frac{var(y-ypred)}{var(y)}$$

where *y* is the measured firing rate, and *ypred* is the firing rate predicted by the model. Proportion of variance explained for predictor *i* was used as a measure of the predictor’s contribution to the model, calculated as:

$${relative\;contribution}_{i}=1-\frac{{\eta^{2}}_{i}}{{\eta^{2}}_{model}}$$

where *η*^2^_*i*_ is the variance explained by the model lacking the predictor *i* (i.e., the weights for predictor *i* are set to zero after training), and *η*^2^_*model*_ is the variance explained by the full model.

Neurons were clustered by their encoding weights using k-means clustering with the number of clusters k determined by maximizing the adjusted Rand Index (ARI), a measure of clustering stability, as a function of number of clusters. We first removed all zero vectors (corresponding to cells that were not explained by the task variables), then computed ARI as the average similarity of 500 pairwise comparisons of independent clusterings of the encoding weights in a given epoch, for k = 2 to 10 clusters. In order to compare stability of clusters across epochs, we chose to use a constant number of clusters across epochs, so we pooled the ARI across epochs to find the peak of the mean curve as a function of k. This gave an optimal k of 6 for clustering cells with non-zero weight vectors, then for the sake of comparison between epochs, we added back the final “cluster” of zero weight vector cells for that epoch to make a total of 7 clusters per epoch.

Comparison of clustering similarity across epochs was measured using the ARI as a measure of pairwise similarity of the clustering between pairs of epochs. This similarity was computed including the cells with zero weight vectors.

### Decoding (linear classifier)

Population activity at a given time point was expressed as a vector of mean rates over a 100-ms bin centered at the time point of interest, for all units recorded on a given session. To estimate the timecourse of activity, activity in 100-ms sliding bins were calculated every 10 ms. To visualize activity trajectories over the trial, principal components decomposition was applied to the population activity matrix, and the activity was projected onto the first 3 principle components.

To assess the amount of information available about a given task variable in the population activity for downstream readout, we trained a linear classifier using the Matlab function *fitclinear* with 5-fold cross validation and lasso regularization on the activity patterns and task variable labels from 90% of valid trials (more below), and assessed the accuracy of predictions on the held out 10% of trials. We optimized the regularization parameter λ by training a 5-fold cross-validated model 30 times using a range of λ values between 0.001 and 0.316, and then selecting the value that produced the lowest cross validation error averaged over all time bins and over runs. We then repeated this modeling 100 times to assess stability of the trained models. We trained the classifier independently at each time point, and then compared the learned weights across time points and across models. The weights were highly consistent across trained models at a given time point, but varied for a given neuron over the course of a trial.

Valid trials were defined as trials on which subjects completed the full trial (through stimulus presentation and the poststimulus delay). To assess choice decoding, we further restricted the trials used to difficult trials, where stimulus discriminability was low and choice profiles approached chance. To assess stimulus decoding, we used trials where the easiest stimuli were presented, to facilitate a one-to-one comparison between the 2 tasks. To correct for the stimulus-choice correlation that existed in the visual decision task (but not in the visually independent auditory task, S3B Fig), we subtracted from the stimulus-decoding accuracy at each time point a choice-decoding correction factor calculated as follows. We calculated the classification accuracy of the stimulus-trained decoder at predicting choice labels on difficult trials, using the same number of difficult trials as the stimulus test set, randomly drawn from the full set of difficult trials on each model repeat. Thus, the performance of the model that was due to actually decoding choice was removed by subtracting the mean accuracy of choice decoding on the correction set, leaving “true stimulus” decoding.

To assess whether the accuracy on the test set was significantly different from chance at a given time point, we trained a classifier on shuffled labels relative to the trial-by-trial stimulus activity. By repeating this on 100 shuffles of the data, we established a 95% confidence interval for each time point in each session. A classifier was labeled as significantly more accurate than chance if its test set accuracy exceeded the upper bound of the confidence interval. Comparisons to assess significance were done on a within-session basis to account for any structure arising from the distribution of trials on that session.

### Hierarchical statistics

Hierarchical statistics adjust for potential dependencies between data points that are sampled from the same animal. Because we measured multiple cells and multiple sessions from each animal, but different animals were trained on the 2 tasks in this study, we applied hierarchical methods to our comparisons between these 2 tasks. Specifically, we adapted the methods previously published in ref [30] to test the hypothesis of whether measurements in condition A were greater than those in condition B, using the following U statistic:

$$U=\sum_{i=1}^{n}\sum_{j=1}^{m}S(a_{i},b_{j})$$

where, *a*_*i*_ *… a*_*n*_ are within-animal means from condition A, *b*_*j*_*…b*_*m*_ are within-animal means from condition B, and S is defined as follows:

$$S(a,b)=\left\{ \begin{array}{c} 1,if\;a>b\\ \frac{1}{2},if\;a=b\\ 0,if\;a<b \end{array}\right..$$

Briefly, we computed the U statistic from the within-animal means, and then resampled the data 1,000 times for each animal via bootstrapping. We permuted the labels for each of the animals 200 times for each bootstrap and computed the U-statistic each time. Thus, 200,000 resamplings formed the null distribution, and p-value was computed as the fraction of the null distribution that was greater than the observed U statistic.

## Supporting information

S1 Fig Timing of peak activity over recording dataset. (a) Cross-validated sorting of neurons by peak activity. Mean activity of single units on odd trials is plotted by order of peak activity on even trials. (b) Mean activity patterns of putative multiunits, sorted by peak activity timing. (c) Counts of recorded units with peak in each epoch, normalized by epoch duration. (d) Proportion of recorded units with peak in each epoch, as a proportion of recorded population. (e) Peak activity timing distribution by animal. (f–h) Proportion of single units selective for stimulus (f), choice (g), and outcome (h), per animal. The underlying data for this figure are available for download from 10.17632/5ms7gcb67j.1.
(TIF)

S2 Fig Non-sensory representations in visual cortex neurons. (a) Distribution of choice probabilities in V1 neurons, as measured by the area under a receiver operating curve. (b, c) Example neurons from Fig 2F and 2G, split by choice (b) and outcome (c) for the same visual stimulus. (d) Side-selectivity index of between-port movements is uncorrelated between choice and initiation movements for cells identified as significantly choice-selective (Pearson correlation, r = −0.056, *p* = 0.483). (e, f) Example neurons and proportion of cells that are selective for previous trial choice (e) and previous trial reward (f) during the pre-stimulus period. The underlying data for this figure are available for download from 10.17632/5ms7gcb67j.1.
(TIF)

S3 Fig Population decoding timecourses are influenced by multiple trial parameters. (a) Mean population activity trajectories (bolded color lines) diverge by trial difficulty. Single trial trajectories are shown in gray. (b) “Stimulus” decoding persists in choice epoch due to strong stimulus-choice correlation in trained animals. (c) Outcome epoch decoding is similar between decoding reward vs. punishment and reward vs. missed reward. The underlying data for this figure are available for download from 10.17632/5ms7gcb67j.1.
(TIF)

S4 Fig Behavior on a visually independent decision task depends on tone location, not visual stimulus distribution. (a) Proportion of left (L) and right (R) choices for both animals (AZ091: solid lines; AZ092: dashed lines) during each recording session, separated by visual stimulus identity. (b) Decision accuracy, defined as choosing the same side as the go-tone was presented, remained stably above 90% across all recording sessions in both animals. The underlying data for this figure are available for download from 10.17632/5ms7gcb67j.1.
(TIF)

S5 Fig Characterization of single neuron responses during visually independent decision task. (a) Recording locations. Blue lines each represent the tetrode bundle center in one animal. (b) Cross-validated sorting of neurons by peak activity. Mean activity of single units on odd trials is plotted by order of peak activity on even trials. (c) Example neuron and proportion of neurons selective for previous trial choice. (d) Example neuron and proportion of neurons selective for previous trial reward delivery. The underlying data for this figure are available for download from 10.17632/5ms7gcb67j.1.
(TIF)

S6 Fig Linear encoding model reveals similar single neuron activity profiles between visually guided and visually independent decision tasks. (a) Firing rate distribution of single units recorded in visually independent decision task. (b) Distribution of maximum relative contribution of a single regressor to single neuron activity in the visually independent decision task, by epoch. The same cutoff threshold separating “specialized” from “mixed” neurons as in the visual decision task is shown in shaded regions. (c) Proportions of cells with “specialized” vs. “mixed” selectivity profiles in the visually independent task, as classified using the threshold in (a). (d) Proportion of variance explained by linear encoding model in the visually independent task, across behavioral epochs. (e) Comparison of variance explained by linear model between visual decision task vs. visually independent decision task, across behavioral epochs. Points indicate mean, error bars indicate standard deviation. Median variance explained is slightly, but not significantly, higher in the visual decision task than in the visually independent decision task within each epoch (hierarchical permutation test, see Methods, all *p* > 0.05). (f) Measure of cluster stability (adjusted Rand Index) when clustering single neuron feature encoding profiles between pairs of epochs, compared to stability over independent partitions in the same epoch (diagonal). (g, h) Pairwise correlation structure between regressors in (g) visual decision task and (h) visually independent task. Regressors are as follows: (1) stimulus, (2) choice, (3) reaction time, (4) movement latency, (5) correctness, (6) reward delivery, (7) previous trial last port visited, (8) previous trial choice, (9) previous trial outcome, and (10) previous trial stimulus. The underlying data for this figure are available for download from 10.17632/5ms7gcb67j.1.
(TIF)

S7 Fig Significance testing of population decoding on visually independent task. (a–e) Proportions of sessions with decoding accuracy significantly greater than chance, for (a) stimulus, (b) choice, (c) outcome, (d) previous choice, and (e) previous outcome. (f) Comparison of decoding accuracy for V1 populations between visually dependent and visually independent choice tasks, during the 500 ms of the trial with the best performance on decoding of each task feature. Points (black = visually dependent task, blue = visually independent task) indicate accuracy on single trials. Comparisons by hierarchical statistical methods for all task features were not significant (hierarchical permutation test, see Methods, all *p* > 0.05). The underlying data for this figure are available for download from 10.17632/5ms7gcb67j.1.
(TIF)
