# Supplementary figures and images for: Neurons in the primary visual cortex of freely moving rats encode both sensory and non-sensory task variables
Source: PLoS Biol. 2023 Dec 4;21(12):e3002384. doi: 10.1371/journal.pbio.3002384 (PMC10721203; doi:10.1371/journal.pbio.3002384)

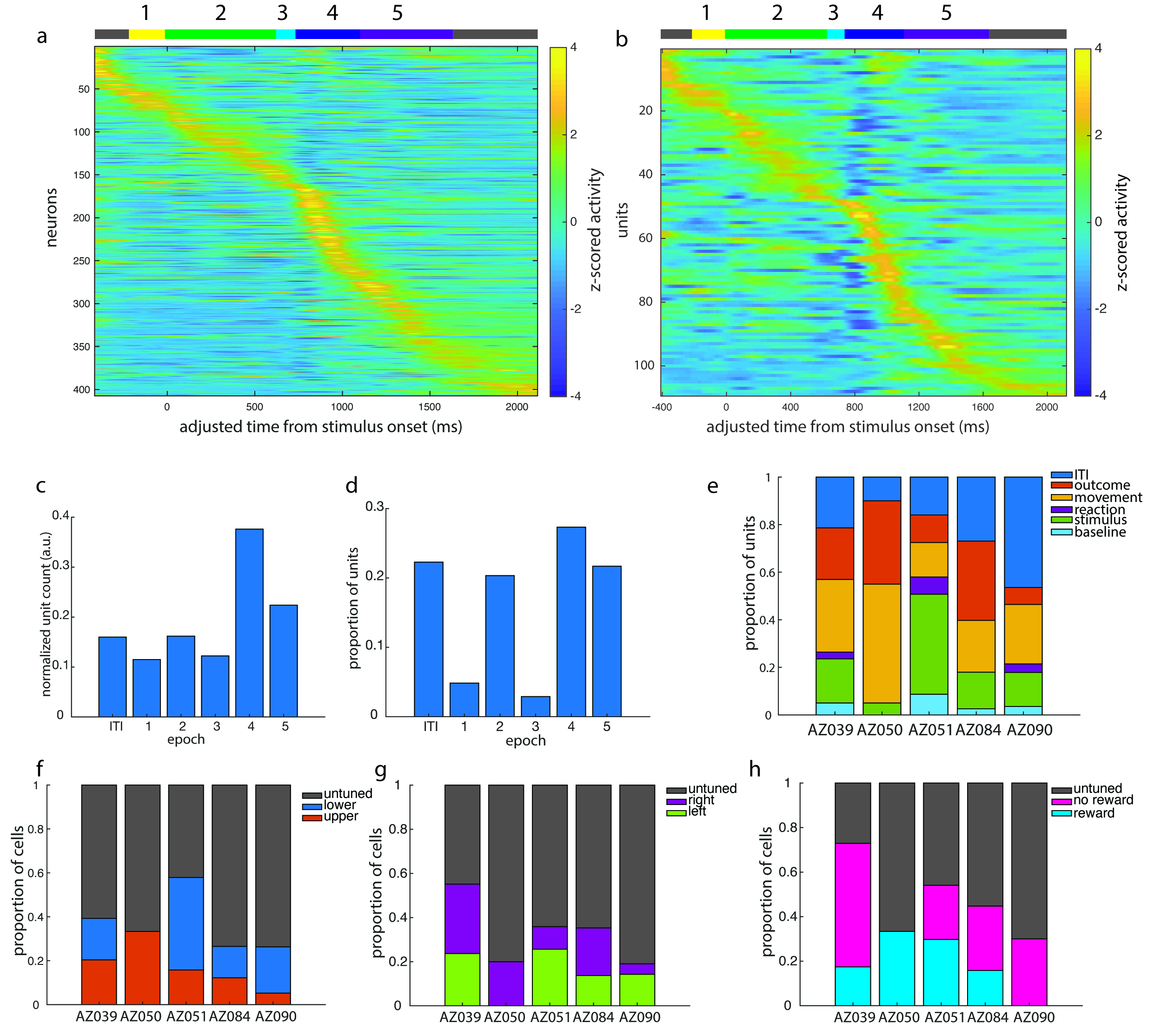

Supplement: S1 Fig — (a) Cross-validated sorting of neurons by peak activity. Mean activity of single units on odd trials is plotted by order of peak activity on even trials. (b) Mean activity patterns of putative multiunits, sorted by peak activity timing. (c) Counts of recorded units with peak in each epoch, normalized by epoch duration. (d) Proportion of recorded units with peak in each epoch, as a proportion of recorded population. (e) Peak activity timing distribution by animal. (f–h) Proportion of single units selective for stimulus (f), choice (g), and outcome (h), per animal. The underlying data for this figure are available for download from 10.17632/5ms7gcb67j.1. (TIF) [file pbio.3002384.s001.tif]

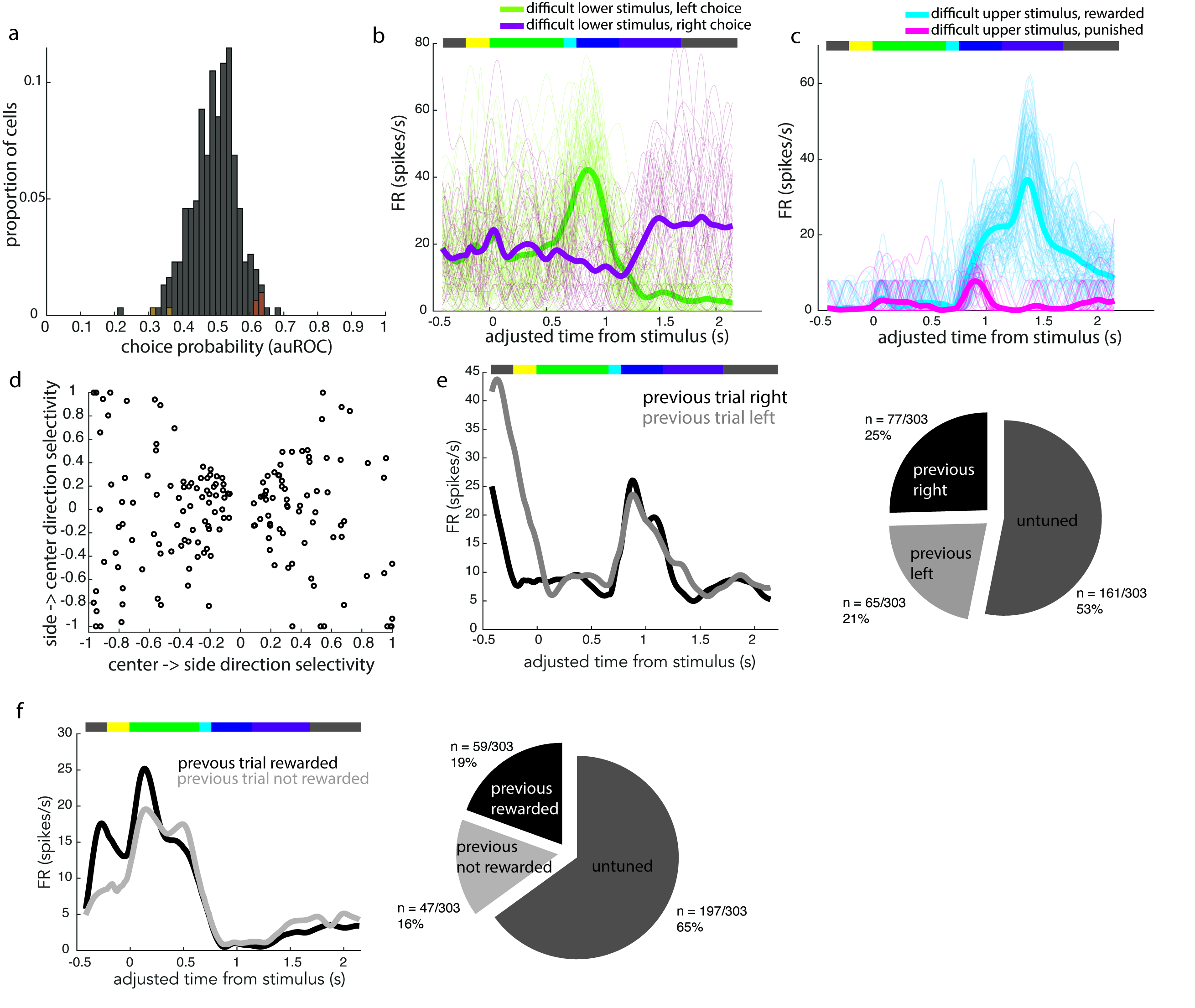

Supplement: S2 Fig — (a) Distribution of choice probabilities in V1 neurons, as measured by the area under a receiver operating curve. (b, c) Example neurons from Fig 2F and 2G, split by choice (b) and outcome (c) for the same visual stimulus. (d) Side-selectivity index of between-port movements is uncorrelated between choice and initiation movements for cells identified as significantly choice-selective (Pearson correlation, r = −0.056, p = 0.483). (e, f) Example neurons and proportion of cells that are selective for previous trial choice (e) and previous trial reward (f) during the pre-stimulus period. The underlying data for this figure are available for download from 10.17632/5ms7gcb67j.1. (TIF) [file pbio.3002384.s002.tif]

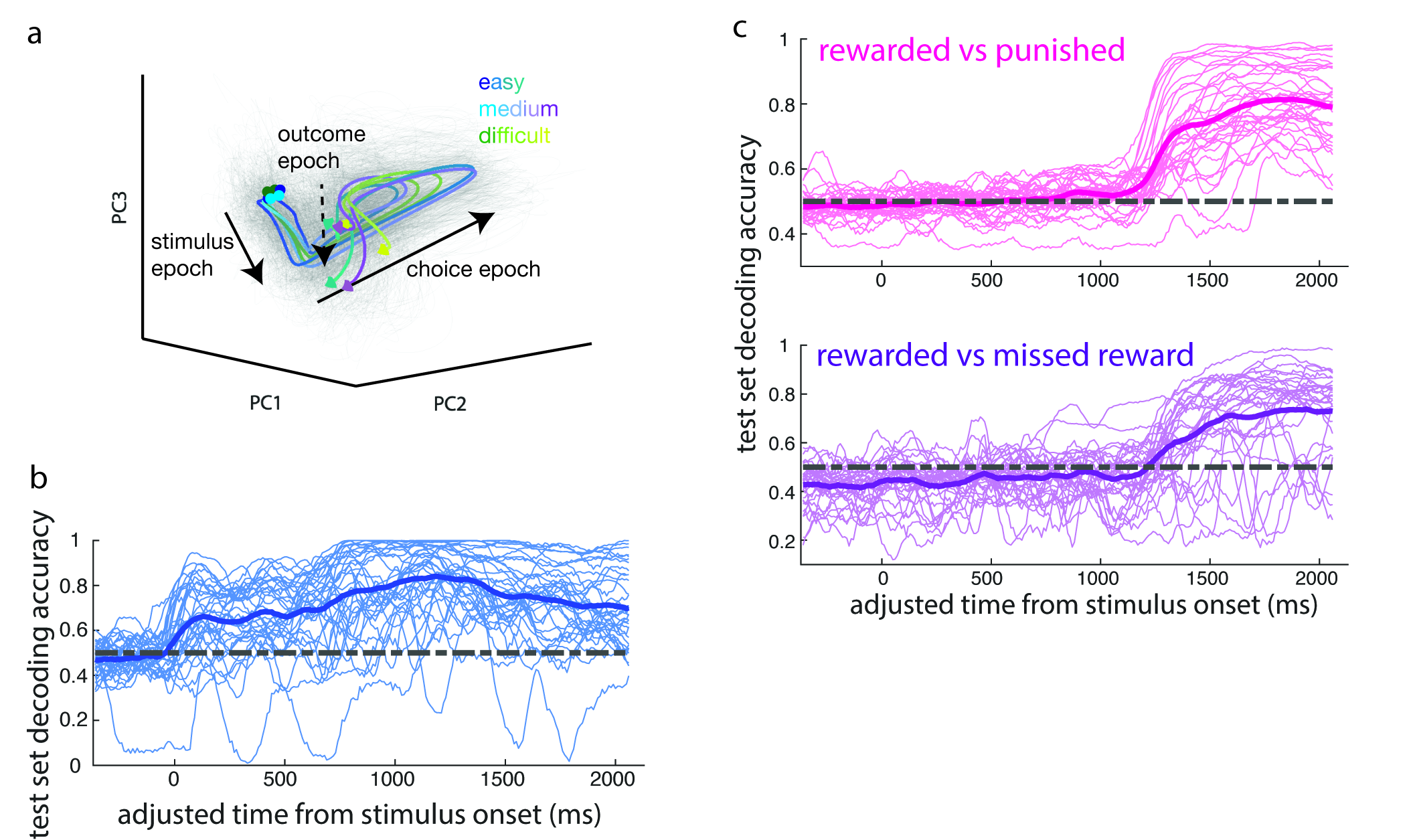

Supplement: S3 Fig — (a) Mean population activity trajectories (bolded color lines) diverge by trial difficulty. Single trial trajectories are shown in gray. (b) “Stimulus” decoding persists in choice epoch due to strong stimulus-choice correlation in trained animals. (c) Outcome epoch decoding is similar between decoding reward vs. punishment and reward vs. missed reward. The underlying data for this figure are available for download from 10.17632/5ms7gcb67j.1. (TIF) [file pbio.3002384.s003.tif]

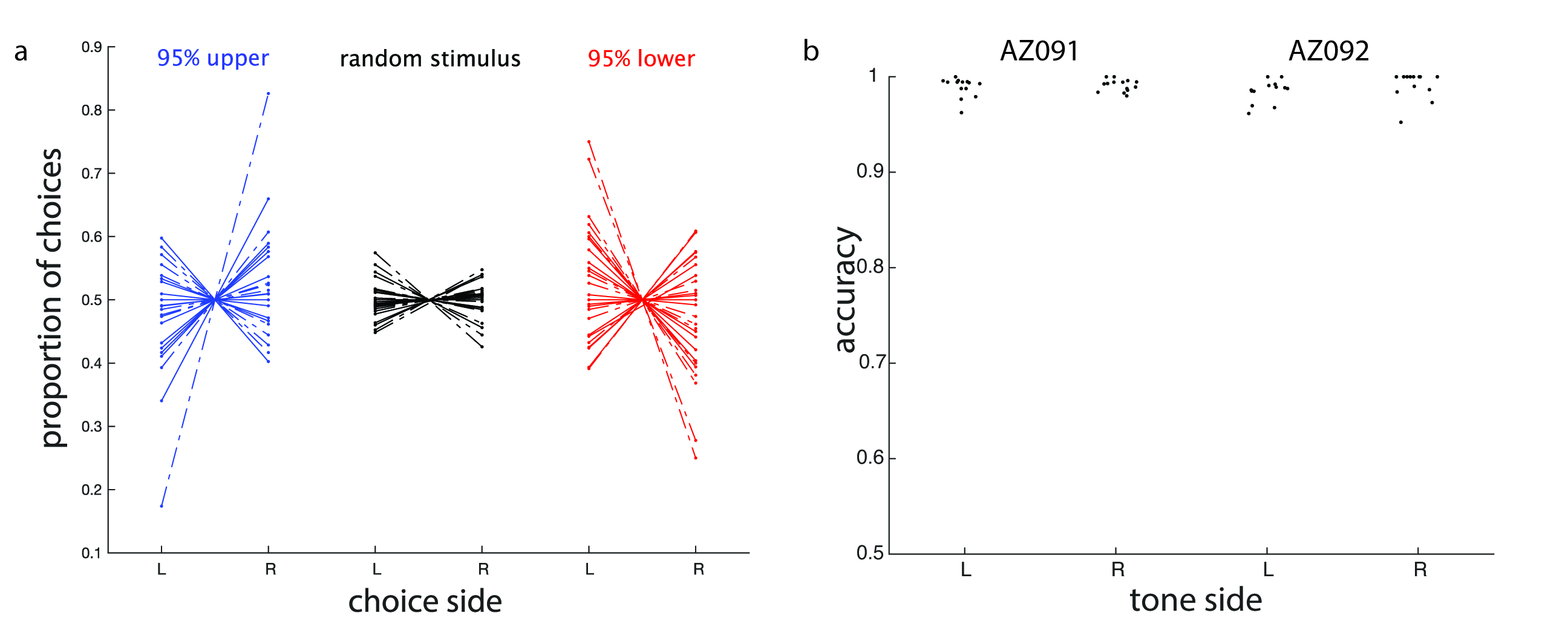

Supplement: S4 Fig — (a) Proportion of left (L) and right (R) choices for both animals (AZ091: solid lines; AZ092: dashed lines) during each recording session, separated by visual stimulus identity. (b) Decision accuracy, defined as choosing the same side as the go-tone was presented, remained stably above 90% across all recording sessions in both animals. The underlying data for this figure are available for download from 10.17632/5ms7gcb67j.1. (TIF) [file pbio.3002384.s004.tif]

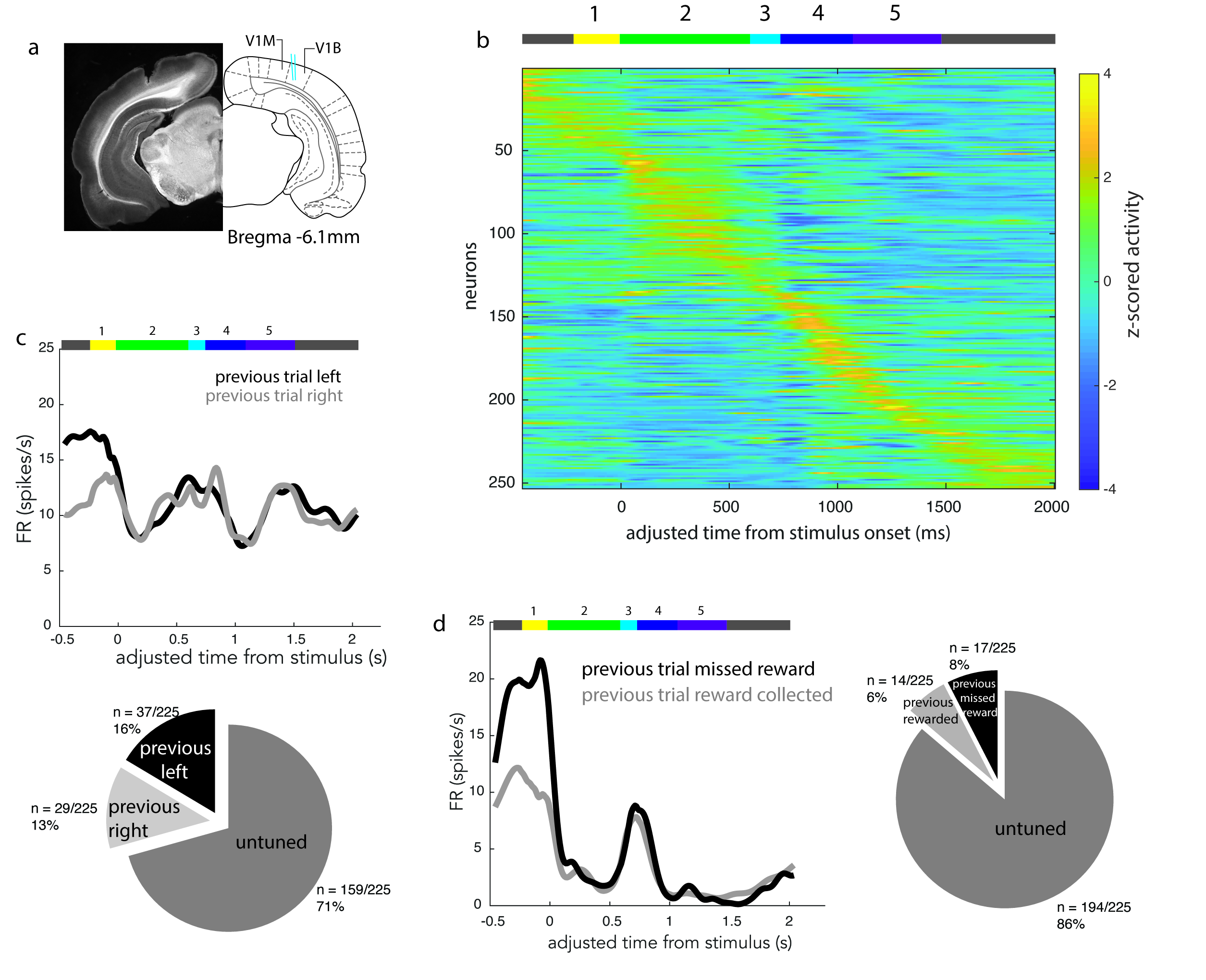

Supplement: S5 Fig — (a) Recording locations. Blue lines each represent the tetrode bundle center in one animal. (b) Cross-validated sorting of neurons by peak activity. Mean activity of single units on odd trials is plotted by order of peak activity on even trials. (c) Example neuron and proportion of neurons selective for previous trial choice. (d) Example neuron and proportion of neurons selective for previous trial reward delivery. The underlying data for this figure are available for download from 10.17632/5ms7gcb67j.1. (TIF) [file pbio.3002384.s005.tif]

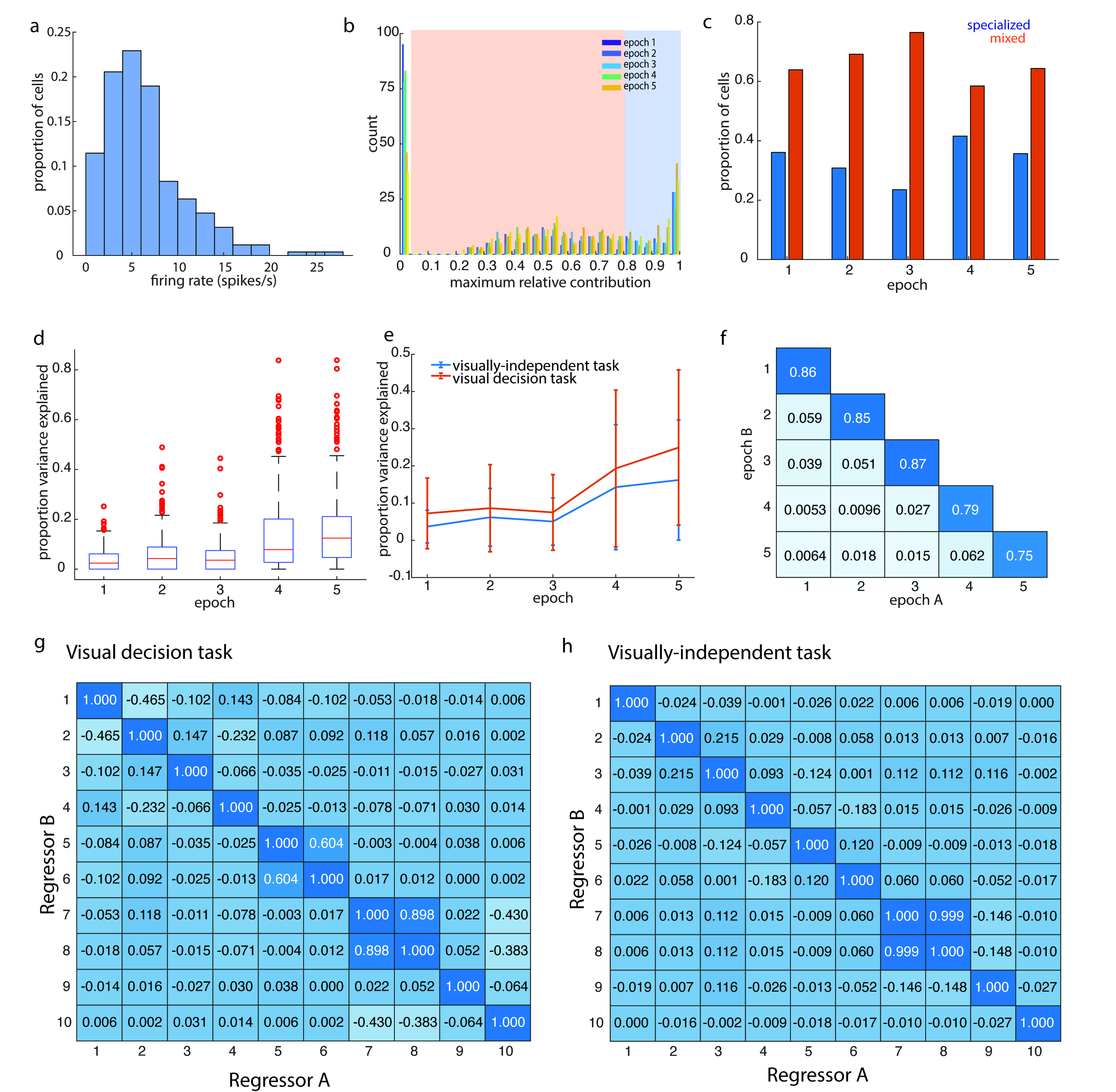

Supplement: S6 Fig — (a) Firing rate distribution of single units recorded in visually independent decision task. (b) Distribution of maximum relative contribution of a single regressor to single neuron activity in the visually independent decision task, by epoch. The same cutoff threshold separating “specialized” from “mixed” neurons as in the visual decision task is shown in shaded regions. (c) Proportions of cells with “specialized” vs. “mixed” selectivity profiles in the visually independent task, as classified using the threshold in (a). (d) Proportion of variance explained by linear encoding model in the visually independent task, across behavioral epochs. (e) Comparison of variance explained by linear model between visual decision task vs. visually independent decision task, across behavioral epochs. Points indicate mean, error bars indicate standard deviation. Median variance explained is slightly, but not significantly, higher in the visual decision task than in the visually independent decision task within each epoch (hierarchical permutation test, see Methods, all p > 0.05). (f) Measure of cluster stability (adjusted Rand Index) when clustering single neuron feature encoding profiles between pairs of epochs, compared to stability over independent partitions in the same epoch (diagonal). (g, h) Pairwise correlation structure between regressors in (g) visual decision task and (h) visually independent task. Regressors are as follows: (1) stimulus, (2) choice, (3) reaction time, (4) movement latency, (5) correctness, (6) reward delivery, (7) previous trial last port visited, (8) previous trial choice, (9) previous trial outcome, and (10) previous trial stimulus. The underlying data for this figure are available for download from 10.17632/5ms7gcb67j.1. (TIF) [file pbio.3002384.s006.tif]

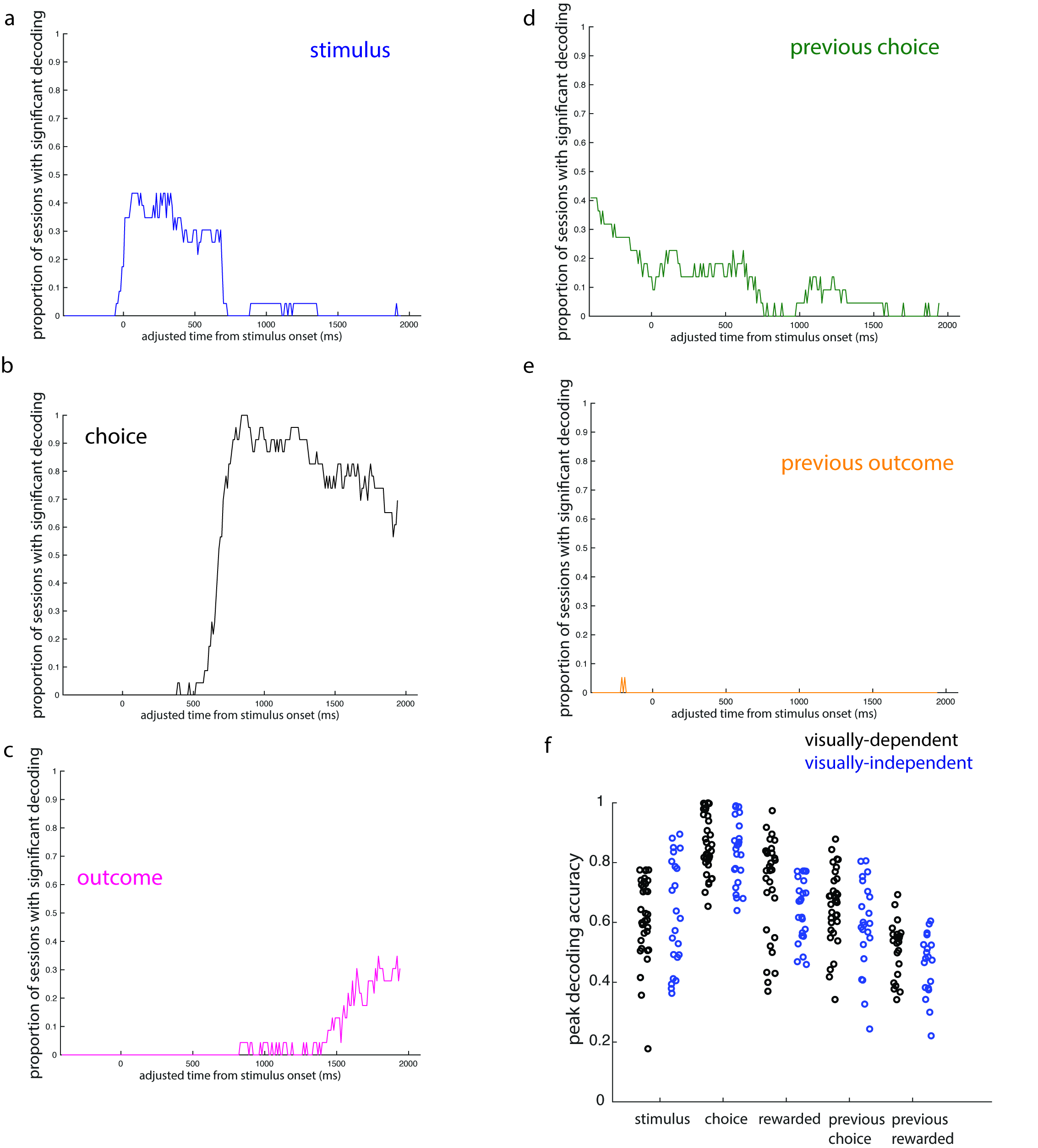

Supplement: S7 Fig — (a–e) Proportions of sessions with decoding accuracy significantly greater than chance, for (a) stimulus, (b) choice, (c) outcome, (d) previous choice, and (e) previous outcome. (f) Comparison of decoding accuracy for V1 populations between visually dependent and visually independent choice tasks, during the 500 ms of the trial with the best performance on decoding of each task feature. Points (black = visually dependent task, blue = visually independent task) indicate accuracy on single trials. Comparisons by hierarchical statistical methods for all task features were not significant (hierarchical permutation test, see Methods, all p > 0.05). The underlying data for this figure are available for download from 10.17632/5ms7gcb67j.1. (TIF) [file pbio.3002384.s007.tif]
